# A Two-Stage Framework for Sensor Selection and Geolocation for eVTOL Emergency Localization Using HF Skywaves

**DOI:** 10.3390/s25247534

**Published:** 2025-12-11

**Authors:** Xijun Liu, Houlong Ai, Chen Xu, Zelin Chen, Zhaoyang Li

**Affiliations:** College of Aviation Electronic and Electrical Engineering, Civil Aviation Flight University of China, Chengdu 641400, China; liuxijun@cafuc.edu.cn (X.L.); ahlcafuc@126.com (H.A.); czlcafuc@126.com (Z.C.); lzy2024@cafuc.edu.cn (Z.L.)

**Keywords:** eVTOL emergency geolocation, high-frequency (HF), non-line-of-sight (NLOS), random spatial spectrum (RSS), receiver selection

## Abstract

High-Frequency (HF) geolocation is crucial for emergency search and rescue operations and for re-geolocation of missing targets. This paper proposes a two-stage (Receiver selection then geolocation with Random Spatial Spectrum (RSS)) framework with HF skywave propagation as the main link, which is suitable for scenarios where the electric Vertical Take-off and Landing (eVTOL) aircraft loses contact, crashes, or has communication interruption after a malfunction. First, stage A employs two receiver selection paths. One is selection with unknown biases, which combines geometric observability to determine receiver selection. The other is selection with bias priors, which introduces non-line-of-sight bias priors and robust weighting to improve availability. Secondly, stage B constructs RSS-based geolocation using grid objective function matching to alleviate the sensitivity of explicit time difference estimation to noise and model mismatch, thereby maintaining robustness under non-line-of-sight (NLOS) conditions. Finally, simulation and actual measurements demonstrate that the “select first, geolocation later” approach achieves superior overall performance compared to direct geolocation without receiver selection. This study provides a methodological basis and initial field evidence for HF skywave-based emergency eVTOL geolocation.

## 1. Introduction

Electric Vertical Take-off and Landing (eVTOL) aircraft are rapidly developing and becoming widely used in Urban Air Mobility (UAM). However, when flying in complex airspace or extreme weather conditions, they may lose their ability to respond due to electric propulsion system failure, communication failure, energy exhaustion, no-fly zones, or obstacles, causing them to crash in unknown geolocation, resulting in loss of geolocation and communication [1,2,3]. Existing emergency search and rescue systems typically rely on 406 MHz CO-SPAS-SARSAT emergency beacons and airborne or ground-based surveillance networks, such as ADS-B, cellular/satellite communications, to successfully complete the final geolocation. Re-geolocation may be delayed or have reduced gain in situations such as beacon failure, antenna proximity, high sea level, or geolocation obstruction [4,5]. High-Frequency (HF, 3–30 MHz) skywaves can propagate beyond the horizon via ionospheric skywaves, covering thousands of kilometers. They can provide a certain level of signal strength in areas with weak ground and satellite coverage or in disaster-stricken areas. As a good geolocation method, they can be used to narrow down aircraft failures such as crashes or forced landings, thereby improving the timeliness of search and rescue [6,7,8]. By utilizing these HF skywaves for passive geolocation, ground monitoring networks can quickly search the area and guide emergency response teams, significantly reducing rescue time. In this context, achieving accurate HF geolocation is crucial for eVTOL search and rescue missions after a crash.

Passive geolocation with HF skywaves has long been a critical capability in applications such as spectrum monitoring, emergency search-and-rescue, signal intelligence, and maritime surveillance [9]. Unlike very-high-frequency (VHF) or microwave bands, HF waves propagate via ionospheric skywave paths, often undergoing one or more reflections before reaching ground-based receivers [10]. This property allows HF signals to cover thousands of kilometers but also introduces substantial uncertainties in their propagation paths, delays, and angles of arrival [11]. Accurate geolocation under such complex multipath and multi-hop conditions is crucial for achieving timely situational awareness and interference source mitigation in modern electromagnetic environments [12,13,14].

Current research on HF passive geolocation mainly follows two technical routes. The first is based on Time Difference of Arrival (TDOA) measurements, where hyperbolic multilateration is used to estimate the emitter’s geolocation from synchronized multi-receiver observations [15,16,17]. Osypiuk, R. [18] enhanced MLAT algorithms by incorporating altitude data alongside TDOA measurements, improving geolocation accuracy and enabling optimized station deployment through nonlinear optimization analysis. Hybrid measurement-based indoor geolocation methods integrating advanced optimization algorithms can mitigate multipath and non-line-of-sight (NLOS) effects, improving geolocation accuracy, robustness, and convergence efficiency in complex environments [19,20]. However, such approaches rely on accurate estimation of individual delays, which becomes highly unreliable in low signal-to-noise ratio (SNR) or multipath conditions. The second route employs Direct Position Determination (DPD) or coherence-based spatial spectrum methods, which bypass explicit TDOA estimation by directly matching the received signals in the time–frequency domain [21,22,23,24]. Ma, F. [25] proposed a TDOA-based DPD method for asynchronous sensor networks that uses anchor sources to jointly calibrate clock biases and estimate source position via Gauss–Newton, achieving accurate localization with reduced computational load. Li, B. [26] introduced a DPD-based Non-homogeneous Data Fusion and Fast Position Update (NDFFPU) algorithm for tracking multiple unknown emitters with distributed arrays, achieving higher accuracy and lower complexity than conventional methods. Ma, F. [27] proposed a particle-filter-based DPD method for moving sources that directly estimates positions and velocities from received signals, achieving improved computational efficiency and estimation accuracy over traditional TDOA/FDOA localization. Weiss, A.J. [28] showed that a single-step DPD method for localizing a stationary transmitter with moving receivers outperforms conventional TDOA/FDOA geolocation in both efficiency and weak-signal accuracy, and derived compact Cramér–Rao lower bound (CRLB) expressions. Eshkevari, A. [29] improved DPD-based localization of co-channel transmitters by introducing a dynamic sensor array response (DSAR) model that accounts for range-dependent path loss, reducing spurious pseudo-spectral-function (PSF) peaks for minimum variance distortionless response (MVDR) and multiple signal classification (MUSIC) beamformers. Xie, J. [30] proposed a despreading DPD (DS-DPD) method for localizing Global Navigation Satellite System (GNSS) spoofers, which jointly exploits delay, Doppler, DOA and code sequence information to achieve order-of-magnitude accuracy gains even at very low interference-to-noise ratio (INR).

However, most of the above studies consider either receiver-geometry optimization or direct geolocation in isolation, rather than combining them in a unified framework. At the same time, the rapid development of the low-altitude economy is leading to an increasing number of eVTOL operations in mountainous and sparsely populated areas where satellite and terrestrial communication coverage can be weak or unavailable. When eVTOL vehicles suffer a serious malfunction or crash in such environments, quickly geolocating the aircraft to support emergency search-and-rescue becomes a critical task. To the best of our knowledge, existing HF skywave geolocation works have not explicitly addressed this eVTOL emergency-rescue scenario. There is also no complete solution that combines the receiver selection phase with the geolocation phase. These considerations motivate the two-stage “select-first, geolocation later” framework studied in this paper.

This paper proposes a two-stage ReSL-RSS (Receiver selection then geolocation with Random Spatial Spectrum (RSS)) framework (Stage A: receiver selection; Stage B: direct geolocation). This technology is designed for eVTOLs flying in low-altitude mountainous areas with sparse populations and no satellite geolocation signals. It also addresses emergency search and rescue scenarios for eVTOLs in situations where communication is interrupted due to connection loss, crashes, or malfunctions.

(1) Stage A employs two receiver selection routes. Case 1 (selection with unknown biases) combines geometric observability to rank and select candidate receivers without relying on prior biases while maintaining the consistency of reference receiver selection. Case 2 (selection with bias priors) builds on this foundation by applying prior constraints and robust weighting on NLOS biases, while also imposing spatial diversity conditions to improve availability and stability in obscured and low-SNR environments.
(2) Stage B adopts an RSS-based direct HF skywave geolocation method centered on grid-based objective function matching over both geographic positions and virtual ionospheric heights. Different from conventional TDOA multilateration or DPD-type methods, the proposed RSS stage operates directly on synchronized multi-receiver I/Q samples and does not estimate any intermediate TOA or TDOA features, which makes the overall ReSL-RSS framework more robust to low SNR, ionospheric model uncertainty and NLOS-affected receivers.
(3) Simulations and actual measurements are performed under the same settings. First, RSS geolocation is performed using receivers from stage A and random receiver selection. Then, the optimal receiver combination output by stage A is determined, simulated and compared with other algorithms, and finally actual measurements are performed.

The results show that, compared with other geolocation algorithms, this two-stage framework can consistently achieve lower geolocation root mean square error (RMSE), smaller median error, more concentrated error distribution, and fewer large error outliers.

## 2. System Model

We consider a passive HF geolocation scenario with a fixed unknown transmitter and multiple time-synchronized receivers located on the Earth’s surface. All coordinates are expressed in geodetic form latitude–longitude. Let the transmitter’s unknown location be $\phi ={\left[\varphi ,\;\lambda \right]}^{⊤}$, where φ is the latitude and λ is the longitude. For each receiver *m* (with m=0,1,…,M−1), we denote its known position by $\xi_{m}={\left[\varphi_{m},\;\lambda_{m}\right]}^{⊤}$. By convention, receiver m=0 serves as the reference sensor. All receivers are assumed to share a common time base and sampling rate $f_{s}$, meaning they collect synchronized baseband in-phase/quadrature (I/Q) samples at sampling rate $f_{s}$. To avoid aliasing and preserve timing accuracy, $f_{s}$ is chosen to be at least twice the occupied signal bandwidth.

In the HF band, skywave propagation causes the transmitted signal to reach each receiver via one or more reflections from the ionosphere. Using the electromagnetic image method, a two-hop propagation path can be treated as an equivalent one-hop path but with an extended propagation delay. Consequently, the overall channel propagation to receiver *m* can be characterized by a sparse discrete impulse response consisting of a few distinct propagation paths. In HF skywave propagation, these effective paths can be further described by an equivalent single-hop virtual-height model. Specifically, the virtual reflection height *h* is constrained to a physically reasonable range of 80–500 km, which covers typical mid-latitude E/F-layer virtual reflection heights for HF links reported in [6,11,12,13]. In our framework, *h* is discretized on a grid and jointly searched together with the source position in Stage B, so that ionospheric variability is treated as an additional model parameter rather than as a fixed deterministic profile.

**Figure 1:**
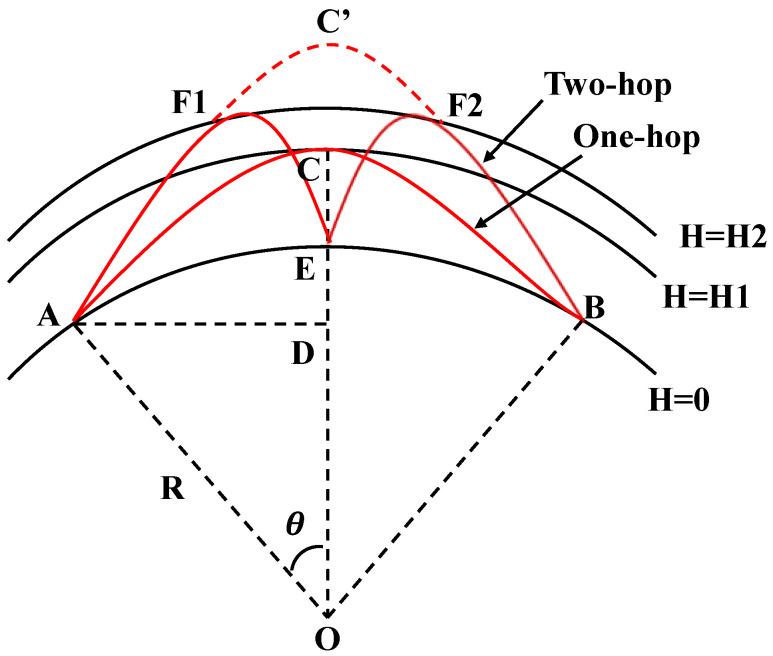
Schematic diagram of HF skywave source geolocation.

In Figure 1, high-frequency signals reach their destination via a single reflection and/or multiple reflections, where the reflection height is obtained by a similar triangular path [31]. According to electromagnetic mirror theory, the two-hop path A–F1–E–F2–B is equal to the path A–C–B, and can also be modeled using a single-hop propagation mode with a longer propagation delay.

We model this impulse response as:

(1) $$g_{m}\left(n\right)\;=\;\sum_{j=0}^{J-1}\beta_{m,j}\;\delta \;\left(n-\tau_{m,j}\right).$$

In Equation (1), *n* is the discrete time index and *J* is the number of significant paths, with path index j=0,1,…,J−1, and we take j=0 to correspond to the direct one-hop path. The coefficient $\beta_{m,j}\in C$ is the complex gain of path *j* for receiver *m*, encompassing the path’s attenuation and phase shift, and $\tau_{m,j}$ is the sample delay (travel time, in samples) for that path from the transmitter to receiver *m*. The delta function δ(·) indicates that each path contributes an impulse at delay $\tau_{m,j}$. Thus, Equation (1) expresses that the discrete-time channel impulse response at sensor *m* can be regarded as a superposition of *J* delayed impulses, each corresponding to one propagation path and weighted by a complex amplitude, which physically leads to a received signal composed of *J* delayed replicas of the transmitted signal.

To compute the geometric propagation delay $\tau_{m,j}$ in Equation (2) for a given path, we consider a spherical Earth model with a virtual ionospheric reflection height for that path. Let $R_{e}$ denote the Earth’s radius, *c* the speed of light, $h_{m,j}$ the virtual ionospheric height associated with path *j* to receiver *m*, and let $\theta_{m}$ be the central angle (at the Earth’s center) between the transmitter’s location ϕ and the receiver’s location $\xi_{m}$. Then the propagation delay in samples for path *j* at receiver *m* is given by:

(2) $$\tau_{m,j}\;=\;\frac{2\;f_{s}}{c}\;\sqrt{\;R_{e}^{2}\sin^{2}\;\theta_{m}\;+\;{\left(R_{e}-R_{e}\cos\;\theta_{m}+h_{m,j}\right)}^{2}},$$

where $\theta_{m}$ in Equation (2) is related to the great-circle distance between transmitter and receiver on the Earth’s surface. The term $R_{e}^{2}\sin^{2}\;\theta_{m}$ corresponds to the squared horizontal distance (across the Earth’s surface) between the two locations, while $\left(R_{e}-R_{e}\cos\theta_{m}+h_{m,j}\right)$ represents the effective vertical path length (difference in radial distance) accounting for the signal’s reflection at height $h_{m,j}$ above the Earth. The factor $2f_{s}/c$ in front converts the physical propagation time to a number of samples, since $f_{s}$ is in samples/second.

The central angle $\theta_{m}$ between the transmitter at ϕ=(φ,λ) and receiver *m* at $\xi_{m}=\left(\varphi_{m},\lambda_{m}\right)$ can be obtained via the spherical law of cosines. In particular, we have

(3) $$\theta_{m}\;=\;\frac{1}{2}\;\arccos\;\left(\sin\varphi_{m}\;\sin\varphi \;+\;\cos\varphi_{m}\;\cos\varphi \;\cos\left(\lambda_{m}-\lambda \right)\right)\;,$$

where $\varphi_{m}$ and $\lambda_{m}$ in Equation (3) are the latitude and longitude of receiver *m*, and φ, λ are the latitude and longitude of the transmitter. Inside the arccos, $\sin\varphi_{m}\;\sin\varphi +\cos\varphi_{m}\;\cos\varphi \;\cos\left(\lambda_{m}-\lambda \right)$ is the spherical dot product of the unit vectors pointing to the two locations, and $\theta_{m}$ is half of the arc-cosine of this quantity. The factor $\frac{1}{2}$ appears because a two-hop path was effectively converted to an equivalent one-hop with extended delay. In essence, $\theta_{m}$ represents the angular separation between transmitter and receiver as seen from the Earth’s center.

Using the delays defined above, we can express the relative arrival times of signals between different receivers. We define the time-difference-of-arrival (TDOA) of path *j* at receiver *m*, relative to the one-hop path at the reference receiver (receiver 0), as

(4) $$\Delta \tau_{m,j}\;=\;\tau_{m,j}-\tau_{0,0}.$$

In other words, $\Delta \tau_{m,j}$ measures how much later in samples path *j* arrives at receiver *m* compared to the reference receiver’s direct path. Using this definition, we can model the discrete-time signal observed at receiver *m* as a sum of delayed copies of the transmitted signal. Specifically,

(5) $$r_{m}\left(t\right)\;=\;\sum_{j=0}^{J-1}\beta_{m,j}\;s\;\left(t-\Delta \tau_{m,j}\right)\;+\;v_{m}\left(t\right)\;,$$

where s(t) in Equation (5) denotes the transmitted signal’s baseband waveform as a function of time index *t*, and $v_{m}\left(t\right)$ represents additive noise at receiver *m*. Equation (5) says that the received signal $r_{m}\left(t\right)$ is composed of *J* copies of s(t), each delayed by $\Delta \tau_{m,j}$ samples and scaled by the complex gain $\beta_{m,j}$, plus noise. In particular, the term for j=0 corresponds to the primary (one-hop) signal component arriving at time $\tau_{0,0}$ at the reference and $\tau_{m,0}$ at receiver *m*, so $\Delta \tau_{m,0}=\tau_{m,0}-\tau_{0,0}$ is the relative delay of the direct path between receiver *m* and the reference.

It is often convenient to examine the signals in the frequency domain. Let *N* be the length of a discrete Fourier transform (DFT) applied to the signals. Denote by S(k) the *k*-th DFT bin of the transmitted signal s(t), and by $R_{m}\left(k\right)$ and $V_{m}\left(k\right)$ the DFTs of $r_{m}\left(t\right)$ and $v_{m}\left(t\right)$, respectively. Then the frequency-domain observation at receiver *m* can be written as

(6) $$R_{m}\left(k\right)\;=\;\sum_{j=0}^{J-1}S\left(k\right)\;\beta_{m,j}\;\exp\;\left\{-\;j\;\frac{2\pi k}{N}\;\Delta \tau_{m,j}\right\}\;+\;V_{m}\left(k\right)\;,\;k=0,1,\ldots ,N-1\;.$$

In Equation (6), each path *j* thus contributes a term to $R_{m}\left(k\right)$ that is the transmitted spectrum S(k) scaled by $\beta_{m,j}$ and rotated in phase by an angle $-\frac{2\pi k}{N}\;\Delta \tau_{m,j}$. This phase shift is due to the path’s time delay $\Delta \tau_{m,j}$. The additive noise in the frequency domain is $V_{m}\left(k\right)$. Equation (6) is simply the DFT of (5) and it shows that the effect of a propagation delay in the time domain is to introduce a frequency-dependent phase shift in the frequency domain.

The goal of the geolocation task is to estimate the transmitter’s coordinates $\phi ={\left[\varphi ,\;\lambda \right]}^{⊤}$ directly from the synchronized multi-channel observations ${\left\{r_{m}\left(t\right)\right\}}_{m=0}^{M-1}$, without explicitly estimating any intermediate delays (such as individual TOAs or TDOAs for each path). In later sections, we will achieve this direct localization by evaluating a random spatial spectrum over a grid of candidate transmitter positions (and associated virtual ionospheric heights) and then finding the location that maximizes a resulting joint statistic.

This system model also supports a two-stage processing pipeline (Stage A + Stage B) for improved performance. Stage A is a sensor selection stage, operating on the same pool of *M* candidate receivers and following the above signal model. In Stage A, we algorithmically select a subset $S_{\mathrm{sel}}\subseteq \left\{0,1,\ldots ,M-1\right\}$ of sensors (receivers) and designate one of them as the reference $r^{*}$, with the aim of optimizing the geometry of the network and improving robustness to mixed line-of-sight (LOS) and non-line-of-sight (NLOS) propagation conditions. Stage B then performs the direct localization on the chosen subset $S_{\mathrm{sel}}$. In Stage B, we use only the selected sensors and reference $r^{*}$: we form TDOA observations relative to $r^{*}$ (as in Equations (1)–(6), but now restricted to the chosen subset and using $r^{*}$ as the reference in place of receiver 0) and evaluate a spatial spectrum over candidate locations. Finally, we identify the source location by maximizing this spectrum. The following sections detail the techniques for Stage A (sensor selection) and Stage B (RSS-based direct localization), respectively.

## 3. Stage A: Sensor Selection Algorithm

Consider a set of *S* candidate receivers (sensors), indexed by i=1,2,…,S, with known positions $s_{i}\in R^{3}$. Each position $s_{i}$ could be specified, for example, in Earth-centered coordinates or another convenient 3D coordinate system; the exact coordinate frame is not critical for the selection algorithm. Let $u\in R^{3}$ denote the (unknown) transmitter’s true position in the same coordinate system. In Stage A, we wish to choose a subset of exactly *K* sensors from these *S* candidates, comprising one reference sensor and K−1 ordinary sensors, to be used for geolocation in Stage B. We introduce binary indicator vectors $q\in {\left\{0,1\right\}}^{S}$ and $p\in {\left\{0,1\right\}}^{S}$ to represent the selection of the reference and ordinary sensors, respectively. Specifically, $q_{i}=1$ if sensor *i* is chosen as the reference and exactly one such *i* must be 1, and $p_{i}=1$ if sensor *i* is chosen as one of the ordinary sensors. No sensor can be both reference and ordinary at the same time. These conditions can be written as selection constraints:

(7) $$\sum_{i=1}^{S}q_{i}=1,\;\sum_{i=1}^{S}p_{i}=K-1,p_{i}+q_{i}\leq 1,\forall \;i=1,\ldots ,S,\;p_{i},q_{i}\in \left\{0,1\right\}.$$

The first constraint ensures exactly one reference is selected, and the second ensures exactly K−1 ordinary sensors are selected, so that in total *K* sensors are chosen. And the third constraint prevents any sensor from being counted as both reference and ordinary. Together, these define the feasible set of selections {p,q}. Each sensor *i* when active can provide a time-of-arrival (TOA) measurement of the signal. We model the TOA measured at sensor *i* as

(8) $$\tau_{i}=\tau_{i}^{o}+n_{i}\;,\;$$

with

(9) $$\tau_{i}^{o}=\frac{d_{i}+l_{i}}{c}\;,\;d_{i}\;≜\;{∥\;u-s_{i}\;∥}_{2}.$$

In Equations (8) and (9), $\tau_{i}$ is the observed TOA at sensor *i*, and it is composed of a true (unbiased) propagation time $\tau_{i}^{o}$ plus a noise term $n_{i}$. The term $\tau_{i}^{o}=\frac{d_{i}+l_{i}}{c}$ in Equation (9) represents the nominal TOA in the absence of noise, where $d_{i}={∥u-s_{i}∥}_{2}$ is the true distance between the unknown source *u* and sensor *i* (Euclidean norm of the difference in position vectors) and $l_{i}\geq 0$ is a bias representing excess path length due to NLOS propagation, if the path is strictly line-of-sight, then $l_{i}=0$. The constant *c* is the signal propagation speed, approximately the speed of light. The noise $n_{i}$ accounts for measurement errors or timing noise at sensor *i* and is assumed to be zero-mean Gaussian with variance $\sigma_{i}^{2}$, i.e., $n_{i}∼N\left(0,\sigma_{i}^{2}\right)$.

If we have designated a particular sensor *r* as the reference (meaning $q_{r}=1$ for some *r*), we can form TDOA measurements between each ordinary sensor *i* and the reference *r*. The TDOA between sensor *i* and the reference *r* is defined as $\tau_{ir}=\tau_{i}-\tau_{r}$. Using the model in (8) and (9), this TDOA can be expressed as

(10) $$\tau_{ir}=\tau_{i}-\tau_{r}=\frac{1}{c}\left(\left(d_{i}+l_{i}\right)-\left(d_{r}+l_{r}\right)\right)+\left(n_{i}-n_{r}\right).$$

In Equation (10), $d_{r}$ is the distance from the source to the reference sensor, and $l_{r}$ is the NLOS bias (if any) for the reference sensor’s path. Equation (10) shows that the true (noise-free) TDOA between sensor *i* and *r* is $\frac{1}{c}\left(d_{i}-d_{r}+l_{i}-l_{r}\right)$, and the noise on this TDOA is $\left(n_{i}-n_{r}\right)$. Note that if both *i* and *r* had purely LOS paths (no biases), the TDOA would simply be $(d_{i}-d_{r})/c$ plus noise, reflecting the difference in distance from the source.

We now stack the TDOA measurements from all selected ordinary sensors (relative to the reference) into a single vector for further processing. Let $M=\{\;i:p_{i}=1\;\}$ denote the set of indices of the K−1 selected ordinary sensors, and let *r* be the reference index with $q_{r}=1$. We form the TDOA observation vector $\tau \in R^{K-1}$ as $\tau ={\left[\;\tau_{ir}:i\in M\;\right]}^{⊤}$. Using Equation (10), this can be written in vector form as

(11) τ=h(u,l)+ξ.

In Equation (11) $h\left(u,\;l\right)\in R^{K-1}$ is a deterministic vector-valued function of the source position *u* and the bias vector $l=(l_{i}:i\in M\cup \left\{r\right\})$; specifically, the *i*th component of h(u,l) (for i∈M) is ${\left[h\left(u,l\right)\right]}_{i}=\frac{1}{c}\left(d_{i}-d_{r}+l_{i}-l_{r}\right)$. The term ξ represents the combined measurement noise. Since each $\tau_{ir}$ has noise $n_{i}-n_{r}$, the noise vector can be modeled as $\xi ∼N(0,\;Q_{r})$, where $Q_{r}$ is the (K−1)×(K−1) noise covariance matrix. From Equation (10), we can derive

(12) $$Q_{r}=\mathrm{diag}\left\{\sigma_{i}^{2}:i\in M\right\}+\sigma_{r}^{2}\;11^{⊤}\;,$$

where $1\in R^{K-1}$ in Equation (12) is a vector of all ones. The diagonal entries of $Q_{r}$ are $\sigma_{i}^{2}+\sigma_{r}^{2}$ for each i∈M, and each off-diagonal entry is $\sigma_{r}^{2}$, because the reference’s noise $n_{r}$ appears in all TDOA measurements and is thus a common source of correlation between any pair of TDOAs.

Next, we derive expressions for the Fisher information to guide our sensor selection. We first consider the line-of-sight only case (i.e., assume all biases $l_{i}=0$, meaning all selected sensors including the reference have unobstructed LOS paths). In this case, the only unknown of interest is the source position *u*. We can write the TDOA model Equation (11) in a simplified form τ=h(u)+ξ where ${h\left(u\right)=h\left(u,l\right)|}_{l\equiv 0}$ corresponds to the purely LOS range differences. The Jacobian matrix of the vector function h(u) with respect to the source position *u* is denoted $H_{u}\in R^{(K-1)\times 3}$. The *i*th row of $H_{u}$ (for a selected sensor i∈M) is given by the partial derivative of the *i*th TDOA (relative to *r*) with respect to *u*. From Equation (10), focusing on the LOS part ($l_{i}=l_{r}=0$), we have $\tau_{ir}^{\left(\mathrm{LOS}\right)}=\frac{1}{c}\left(d_{i}-d_{r}\right)$, where $d_{i}={∥u-s_{i}∥}_{2}$ and $d_{r}={∥u-s_{r}∥}_{2}$. Differentiating this with respect to the components of *u*, we obtain:

(13) $$\frac{\partial }{\partial u^{⊤}}\left[\frac{1}{c}\left(d_{i}-d_{r}\right)\right]=\frac{1}{c}\left(\frac{{\left(u-s_{i}\right)}^{⊤}}{∥u-s_{i}{∥}_{2}}-\frac{{\left(u-s_{r}\right)}^{⊤}}{∥u-s_{r}{∥}_{2}}\right)\;,\;\mathrm{for}\;\mathrm{each}\;i\in M,$$

where the row vector on the right-hand side of Equation (13) is exactly the *i*th row of $H_{u}$. Intuitively, $\frac{u-s_{i}}{∥u-s_{i}{∥}_{2}}$ is the unit vector pointing from sensor *i* toward the source or vice versa, and $\frac{u-s_{r}}{∥u-s_{r}{∥}_{2}}$ is the unit vector pointing from the reference *r* toward the source. Thus, the row in Equation (13) is proportional to the difference between these two unit direction vectors, scaled by 1/c. Stacking all selected sensors’ rows, we get the full Jacobian matrix $H_{u}$.

Meanwhile, if we consider the dependence of the TDOA model on the bias terms $l_{i}$, the Jacobian with respect to the bias vector *l* can also be written down. From Equation (10), each ordinary sensor *i*’s TDOA depends on $l_{i}$ with coefficient +1/c, and on the reference’s bias $l_{r}$ with coefficient −1/c. Therefore, the Jacobian with respect to the biases for the selected sensors (including the reference) is

(14) $$H_{l}=\frac{1}{c}\left[\;I_{K-1}\;-1\;\right],$$

where $I_{K-1}$ in Equation (14) is the (K−1)×(K−1) identity matrix with rows corresponding to the ordinary sensors’ biases $l_{i}$ for i∈M, and −1 is a (K−1)×1 column vector corresponding to the derivative with respect to the reference bias $l_{r}$. Thus, each row of $H_{l}$ has a 1/c in the column for its own bias $l_{i}$ and a −1/c in the column for $l_{r}$, indicating that an increase in $l_{i}$ increases $\tau_{ir}$ by 1/c, while an increase in $l_{r}$ decreases all $\tau_{ir}$ by 1/c.

Now we can formulate the Fisher Information Matrix (FIM) for the source localization problem under various assumptions:

Pure LOS case (no biases): In this scenario (l≡0 known), the only unknown parameters are the coordinates of *u*. The FIM for estimating *u*, given a particular choice of sensors (encoded by p,q), is

(15) $$J_{u}^{\mathrm{LOS}}\left(u|p,q\right)=H_{u}^{⊤}\;Q_{r}^{-1}\;H_{u},$$

where this is a 3×3 matrix in Equation (15) quantifies the information (inverse variance) available about the source position *u*. The CRLB for unbiased estimation of *u* under these conditions is given by the inverse of this matrix: ${\mathrm{CRLB}}^{\mathrm{LOS}}\left(u|p,q\right)={\left(J_{u}^{\mathrm{LOS}}\right)}^{-1}$. In practice, one might use a scalar summary of this matrix (for example, the trace or the largest eigenvalue) as a measure of the expected localization error for the chosen sensors.

Unknown bias case (NLOS present, no prior): In this case, the bias values $l_{i}$ for NLOS paths are unknown parameters to be estimated jointly with *u*. We refer to this scenario as Case1 (position and sensor biases unknown, with no prior information on biases). We then have an augmented parameter vector $\theta ={\left[\;u^{⊤},\;l^{⊤}\right]}^{⊤}$ that includes both the source position and all selected bias terms. The FIM for this joint parameter vector (prior to incorporating any prior distributions) can be constructed in block-matrix form using the Jacobians $H_{u}$ and $H_{l}$ derived above:

(16) $$J_{\theta }^{(\mathrm{no}\;\mathrm{prior})}=\left[\begin{array}{cc} H_{u}^{⊤}Q_{r}^{-1}H_{u} & \;H_{u}^{⊤}Q_{r}^{-1}H_{l}\\ H_{l}^{⊤}Q_{r}^{-1}H_{u} & \;H_{l}^{⊤}Q_{r}^{-1}H_{l} \end{array}\right]\;.$$

The top-left block $H_{u}^{⊤}Q_{r}^{-1}H_{u}$ corresponds to information about *u* (as in the pure LOS case), the bottom-right block $H_{l}^{⊤}Q_{r}^{-1}H_{l}$ corresponds to information about the biases *l*, and the off-diagonal blocks $H_{u}^{⊤}Q_{r}^{-1}H_{l}$ (and its transpose) couple the two. Since our ultimate interest is in *u* (the source location), we can obtain an effective Fisher information matrix for *u* alone by marginalizing out (or Schur-complementing) the nuisance parameters *l*. The effective information matrix for *u* in this unknown-bias scenario is given by the Schur complement of the *l*-block in Equation (16):

(17) $$J_{u}^{\mathrm{Case}1}\left(u|p,q\right)=H_{u}^{⊤}Q_{r}^{-1}H_{u}-H_{u}^{⊤}Q_{r}^{-1}H_{l}\;{\left(H_{l}^{⊤}Q_{r}^{-1}H_{l}\right)}^{-1}H_{l}^{⊤}Q_{r}^{-1}H_{u}\;.$$

A practical and often overlooked consequence follows: if the reference is LOS ($l_{r}\;=\;0$), $J_{u}^{\mathrm{Case}1}$ depends only on LOS rows of $H_{u}$; unmodeled NLOS rows do not increase information about *u*. This motivates choosing a reference that is likely LOS and discounting suspected NLOS rows during selection.

Unknown bias case with priors: In some situations, we may have prior knowledge or statistical estimates for the NLOS biases $l_{i}$. For example, from historical data or auxiliary sensors that can gauge NLOS conditions. We refer to this scenario as Case2 (position and sensor biases known a priori, or at least we have prior distributions for them). We can incorporate this prior information in the FIM as additional “information” about the parameters. Suppose we have an independent prior for each bias $l_{i}$ for i∈M∪{r}, with prior variance $w_{i}^{2}$, so $w_{i}$ is the standard deviation of our prior belief about bias $l_{i}$. We can form a diagonal information matrix for these priors as:

(18) $$J_{\mathrm{prior}}=\mathrm{diag}\left(0_{3\times 3},\;\Omega \right)\;,\;\Omega =\mathrm{diag}\left\{w_{i}^{-2}:i\in M\right\}\;⊕\;\left[\;w_{r}^{-2}\;\right]\;.$$

In $J_{\mathrm{prior}}$, the top-left 3×3 block is all zeros because we assume no prior information on the true source position *u*, and the matrix Ω on the diagonal corresponds to the information (inverse variance) we have for each bias: it contains $w_{i}^{-2}$ for each selected ordinary sensor *i* and $w_{r}^{-2}$ for the reference’s bias.

The generalized Fisher information matrix when considering both the data and the bias priors is then the sum of the information from the measurements Equation (16) and the prior information above. Partition $J_{\theta }^{\mathrm{Case}2}$ conformably with $\theta ={\left[u^{⊤},\;l^{⊤}\right]}^{⊤}$ as:

(19) $$J_{\theta }^{\mathrm{Case}2}=\left[\begin{array}{cc} J_{uu} & J_{ul}\\ J_{lu} & J_{ll} \end{array}\right]\;.$$

In Equation (19), $J_{uu}$ is the 3×3 block corresponding to *u*, $J_{ll}$ the K×K block for *l*, and $J_{ul}=J_{lu}^{⊤}$ the cross terms. Then the effective Fisher information for *u* (marginalizing out *l* with its priors considered) is:

(20) $$J_{u}^{\mathrm{Case}2}\left(u|p,q\right)=J_{uu}-J_{ul}\;J_{ll}^{-1}\;J_{lu}\;.$$

This formula is analogous to Equation (17), but now $J_{ll}^{-1}$ incorporates both the measurement information and the prior information about the biases. Because we have some knowledge of the biases (through the prior), even NLOS sensors can contribute some information about *u*, unlike the prior-free case, although the contribution is reduced depending on the uncertainty of the bias (encoded in $w_{i}^{2}$).

With these Fisher information matrices in hand, we can formalize the Stage A sensor selection problem. The goal is to choose *p* and *q* (i.e., choose which sensor is reference and which K−1 are ordinary) to minimize the expected localization error of Stage B. As a metric, one convenient choice is the trace of the position error covariance matrix (the trace of the CRLB). Other criteria, such as the log-determinant of the CRLB or the largest eigenvalue (worst-case axis), could also be used—typically all such criteria will lead to a similar selection.

We formulate two versions of the selection problem corresponding to the scenarios above:

(Case1) Selection with unknown biases: We assume some sensors could be NLOS (unknown biases) and no bias priors are used. We then want to minimize the trace of the position-only CRLB accounting for unknown biases, which is based on $J_{u}^{\mathrm{Case}1}$ from Equation (17):

(21) $$\begin{array}{cc} \left(\mathrm{Case}1\right)\; & \min_{p,q}\;\;\mathrm{trace}\left({\left(J_{u}^{\mathrm{Case}1}\left(u|p,q\right)\right)}^{-1}\right)\\ s.t.\; & \sum_{i=1}^{S}q_{i}=1,\;\sum_{i=1}^{S}p_{i}=K-1,\;p_{i}+q_{i}\leq 1\;\;\forall \;i,\;p_{i},q_{i}\in \left\{0,1\right\}\;. \end{array}$$

(Case2) Selection with bias priors: We assume we have prior information for biases. The selection aims to minimize the trace of the CRLB using $J_{u}^{\mathrm{Case}2}$ from Equation (20):

(22) $$\begin{array}{cc} \left(\mathrm{Case}2\right)\; & \min_{p,q}\;\;\mathrm{trace}\left({\left(J_{u}^{\mathrm{Case}2}\left(u|p,q\right)\right)}^{-1}\right)\\ s.t.\; & \sum_{i=1}^{S}q_{i}=1,\;\sum_{i=1}^{S}p_{i}=K-1,\;p_{i}+q_{i}\leq 1\;\;\forall \;i,\;p_{i},q_{i}\in \left\{0,1\right\}\;. \end{array}$$

Each of these is a combinatorial optimization problem: we are searching over all choices of one reference and K−1 others out of *S* to minimize the chosen objective. The problem can be computationally challenging if *S* is large, since the number of subsets to check grows combinatorially. In practice, one can use heuristic or relaxed optimization methods. For example, one approach is to use a convex relaxation, such as formulating an equivalent semidefinite programming (SDP) problem and then using randomized rounding to obtain a binary solution, to get a near-optimal solution offline. Alternatively, a faster greedy algorithm can be used for online selection: for instance, one can start with an empty set and iteratively add the sensor that most improves the objective greedy forward selection, or start with a random set and then iteratively swap sensors in and out to improve the objective. These algorithms work directly with the information matrices ($J_{u}^{\mathrm{Case}1}$ or $J_{u}^{\mathrm{Case}2}$ as appropriate) and enforce the selection constraints Equation (7) at each step.

In implementing these selection strategies, it is often useful to have an initial rough estimate of the source location *u*. The matrix $H_{u}$ and the resulting Fisher information depend on *u* only through the geometry terms $\left(u-s_{i}\right)/∥u-s_{i}∥$ in Equation (13). In a practical deployment, such a coarse estimate $\hat{u}$ can be obtained directly from the TOA/TDOA measurements used in Stage A: once the HF distress signal is received, one receiver is chosen as a time reference and each active sensor forms a TOA or TDOA measurement according to the model in Section 3. These TDOA values define a family of hyperbolic curves with foci at the receiver positions. By intersecting these hyperbolas once, using all available receivers, we obtain a coarse multilateration solution $\hat{u}$. This simple hyperbolic multilateration ignores NLOS biases and does not iterate, so its accuracy is limited, but it provides a point inside the region of interest. Using this approximate $\hat{u}$ to evaluate $H_{u}$ therefore supplies a representative geometry for the sensor-selection criteria without requiring any accurate prior knowledge of the true eVTOL position.

To summarize the above procedure, the following Algorithm 1 outlines a generic implementation of Stage A for both Case 1 and Case 2.

**Algorithm 1** Stage A: Receiver Selection (Case 1/Case 2)1:**Input:** candidate receivers ${\left\{s_{i}\right\}}_{i=1}^{S}$ with positions $\xi_{i}$; approx. source $\hat{u}$; selection budget *K* (one reference $r^{★}$ and K−1 ordinary); noise covariance $Q_{r}$; (Case 2) bias prior variances $\left\{w_{i}^{2}\right\}$; mode mode∈{Case1,Case2}; optional diversity constraints.
2:**Output:** selected subset $S_{\mathrm{sel}}$ with $|S_{\mathrm{sel}}|=K$, including reference $r^{★}$.
3:Using $\hat{u}$ and $\left\{\xi_{i}\right\}$, construct the Jacobians $H_{u}\left(\hat{u}\right)$ and $H_{l}\left(\hat{u}\right)$ and build the Fisher information blocks (Section 3).
4:Define a function FIM(p,q) that, for any feasible binary selection (p,q) satisfying $\sum_{i=1}^{S}q_{i}=1$, $\sum_{i=1}^{S}p_{i}=K-1$, $p_{i}+q_{i}\leq 1$, $p_{i},q_{i}\in \left\{0,1\right\}$, returns the position-only FIM $J_{u}^{\mathrm{Case}\;1}\left(\hat{u}\;|\;p,q\right)$ if mode=Case1, and $J_{u}^{\mathrm{Case}\;2}\left(\hat{u}\;|\;p,q\right)$ otherwise.
5:$J_{\mathrm{best}}\leftarrow +\infty$;    $(p^{★},q^{★})\leftarrow ⌀$.6:**for all** feasible (p,q) that select exactly one reference and K−1 ordinary receivers and satisfy the diversity constraints **do**
7:   J←FIM(p,q)
8:   $c\left(p,q\right)\leftarrow \mathrm{tr}\;\left({\left(J\right)}^{-1}\right)$ trace of the position-only CRLB}
9:   **if** 
$c\left(p,q\right)<J_{\mathrm{best}}$
 **then**
10:      $J_{\mathrm{best}}\leftarrow c\left(p,q\right)$;   $\left(p^{★},q^{★}\right)\leftarrow \left(p,q\right)$
11:   **end if**12:
**end for**
13:$S_{\mathrm{sel}}\leftarrow \left\{\;i|p_{i}^{★}=1\;\mathrm{or}\;q_{i}^{★}=1\;\right\}$ {chosen *K* receivers}
14:$r^{★}\leftarrow \arg\max_{i}q_{i}^{★}$ {the single reference (the index with $q_{i}^{★}=1$)}
15:**Return:** $S_{\mathrm{sel}}$ and $r^{★}$.


## 4. Stage B: Geolocation Method

Stage B employs a Random Spatial Spectrum (RSS)-based direct localization algorithm that works directly with synchronized HF receiver data under the equivalent single-hop skywave model. Classical DPD [32,33,34] techniques are also single-step estimators that use the same received data as conventional two-step schemes, but they usually assume LOS or NLOS propagation and fixed sensor geometry and construct a global maximum-likelihood or subspace cost function by coherently combining array outputs over candidate emitter locations, sometimes together with delay/Doppler parameters. In contrast, the RSS stage in our framework evaluates a spatial spectrum on a joint grid of ground positions and virtual ionospheric heights by computing cross-spectral coherence terms between the reference and each selected receiver and aggregating them into a joint spatial spectrum F(u). In this way, Stage B does not explicitly estimate any TOA/TDOA or FDOA parameters and remains consistent with the virtual-height skywave model and the receiver subset determined in Stage A.

Let $S_{\mathrm{sel}}$ be the subset of sensor indices chosen in Stage A, with $|S_{\mathrm{sel}}|=K$. Denote the reference sensor chosen by Stage A as $r^{★}\in S_{\mathrm{sel}}$, and let $M=S_{\mathrm{sel}}\setminus \left\{\;r^{★}\;\right\}$ be the set of the remaining K−1 selected sensors (the ordinary sensors). By construction, |M|=K−1. For each selected sensor $m\in S_{\mathrm{sel}}$, we have a stream of synchronized baseband samples $r_{m}\left(t\right)$ (as described in the system model). We can transform these to the frequency domain; let $K_{f}$ denote the length of the DFT we apply (this could be equal to *N* used in Stage A or a different value as needed), and let $K\subseteq \{0,1,\ldots ,K_{f}-1\}$ be the set of frequency bins we will use for localization (for example, we might exclude very low-frequency bins or very noisy bins). Then for each selected sensor *m*, we have frequency-domain data $R_{m}\left(k\right)$ for k∈K.

Stage B proceeds by evaluating an RSS on a grid of candidate source positions and possible path heights, then finding the maximizer of this spectrum as the estimated source location. We define the search grid as follows:

- Let $U=\left\{\;u^{\left(p\right)}:p=1,2,\ldots ,P\;\right\}\subset R^{3}$ be a set of *P* candidate source positions (particles) that we will test. These could be, for example, points on or near the Earth’s surface in the geographic region of interest (forming a latitude–longitude grid, possibly with some altitude dimension if altitude is uncertain).
- Let $H=\left\{\;h^{\left(q\right)}:q=1,2,\ldots ,Q\;\right\}\subset R_{+}$ be a set of *Q* candidate virtual ionospheric heights to consider for the signal paths. This grid allows the algorithm to consider different possible effective heights of reflection for the paths.

With these grids, the RSS algorithm works as follows.

Path-delay computation for candidates: For any hypothesized source position u∈U and any hypothesized virtual reflection height h∈H, we can compute the expected propagation delay (in samples) from *u* to sensor *m* by an equation analogous to (2). Namely,

(23) $$\tau_{m}\left(u,h\right)=\frac{2\;f_{s}}{c}\;\sqrt{\;R_{e}^{2}\;\sin^{2}\;\Theta_{m}\left(u\right)\;+\;{\left(R_{e}-R_{e}\cos\;\Theta_{m}\left(u\right)+h\right)}^{2}}\;.$$

In Equation (23), $R_{e}$ is the Earth’s radius (same as before), and $\Theta_{m}\left(u\right)$ represents the central angle (at Earth’s center) between the candidate position *u* and sensor *m*’s location $s_{m}$. This $\Theta_{m}\left(u\right)$ can be computed similarly to $\theta_{m}$ in (3), except that now *u* may be a variable point not equal to the true source. Throughout the paper, $\theta_{m}$ is reserved for the central angle associated with the (unknown) true source position *u* in the channel model of Equations (2) and (3), whereas $\Theta_{m}\left(u\right)$ denotes the central angle associated with a hypothesized candidate location *u* when evaluating the Stage B grid. Essentially, $\tau_{m}\left(u,h\right)$ is the modeled propagation delay (in samples) from the candidate source at *u* to sensor *m*, assuming a single-hop path that reaches an altitude of *h* before coming down to *m*. (This formulation also effectively models multi-hop paths by using an equivalent single-hop with a larger *h*, just as we did in Stage A).

Using $\tau_{m}\left(u,h\right)$, we can generate hypothesized TDOAs for each pair of paths (one for sensor *m* and one for the reference $r^{★}$) as follows. For each ordered pair of heights $(q,q^{\prime })\in H\times H$, define

(24) $$\Delta \tau_{m}^{\left(q,q^{\prime }\right)}\left(u\right)=\tau_{m}\;\left(u,\;h^{\left(q\right)}\right)-\tau_{r^{★}}\;\left(u,\;h^{\left(q^{\prime }\right)}\right)\;,\;\mathrm{for}\;\mathrm{each}\;\;m\in M.$$

In Equation (24), $\Delta \tau_{m}^{\left(q,q^{\prime }\right)}\left(u\right)$ is a hypothesized time-difference-of-arrival: it assumes that the signal to sensor *m* traveled via an ionospheric layer of height $h^{\left(q\right)}$, and the signal to the reference $r^{★}$ traveled via height $h^{\left(q^{\prime }\right)}$. By considering all combinations $\left(q,q^{\prime }\right)$, we are effectively allowing the possibility that the path to *m* might involve a different number of hops (or a different reflection height) than the path to the reference. In HF propagation, for instance, one receiver might receive a one-hop signal while another simultaneously receives a two-hop signal from the same transmitter; including both *q* and $q^{\prime }$ in the grid allows the algorithm to match such situations by trying a larger virtual height for one path.

Per-sensor coherence calculation: Next, for each candidate position *u* and each sensor m∈M, we quantify how well the data from sensor *m* aligns with the data from the reference $r^{★}$ if the transmitter were at *u* with a particular pair of path heights. We do this via a cross-spectral coherence* calculation. Specifically, for a given *u* and a given pair $\left(q,q^{\prime }\right)$, we compute

(25) $$C_{m}\;\left(u;\;q,q^{\prime }\right)\;=\;\frac{1}{\left|K\right|}\sum_{k\in K}\frac{R_{r^{★}}^{H}\left(k\right)\;R_{m}\left(k\right)}{\;|R_{r^{★}}\left(k\right){|}^{2}+\varepsilon \;}\;\exp\;\left\{\;j\;\frac{2\pi k}{K_{f}}\;\Delta \tau_{m}^{\left(q,q^{\prime }\right)}\left(u\right)\right\}.$$

In Equation (25), the term $R_{r^{★}}^{H}\left(k\right)R_{m}\left(k\right)$ is the complex product of the reference’s DFT with the conjugate (denoted by *H* for Hermitian transpose) of sensor *m*’s DFT at frequency bin *k*—essentially the cross-power spectrum between the reference and sensor *m* at that frequency. We then divide by $|R_{r^{★}}\left(k\right){|}^{2}+\varepsilon$, where $|R_{r^{★}}\left(k\right){|}^{2}$ is the power at the reference in that bin; this normalization down-weights frequency bins where the reference signal is very weak (the small positive term ε is added to avoid division by zero or numerical instability when $|R_{r^{★}}\left(k\right){|}^{2}$ is extremely small). Next, we multiply by $\exp\;\left\{j\frac{2\pi k}{K_{f}}\Delta \tau_{m}^{\left(q,q^{\prime }\right)}\left(u\right)\right\}$, which is a phase rotation compensating for the hypothesized TDOA $\Delta \tau_{m}^{\left(q,q^{\prime }\right)}\left(u\right)$ at that frequency. If the guess $\Delta \tau_{m}^{\left(q,q^{\prime }\right)}\left(u\right)$ is correct for the true propagation path difference, this phase factor will align the signal from sensor *m* with the reference’s signal in that frequency bin. Finally, we average over all frequency bins k∈K (hence the $\frac{1}{\left|K\right|}\sum_{k}$) to obtain the coherence measure $C_{m}\left(u;q,q^{\prime }\right)$. In essence, $C_{m}\left(u;q,q^{\prime }\right)$ measures the correlation between sensor *m*’s received signal and the reference’s signal when we time-shift one relative to the other by the hypothesized delay $\Delta \tau_{m}^{\left(q,q^{\prime }\right)}\left(u\right)$. If the transmitter were truly at *u* and the path heights were $h^{\left(q\right)}$ and $h^{\left(q^{\prime }\right)}$ for *m* and $r^{★}$ respectively, we would expect $C_{m}\left(u;q,q^{\prime }\right)$ to be large (its magnitude close to 1, say). If the hypothesis is wrong, the signals will not align well and the magnitude $\left|C_{m}\left(u;q,q^{\prime }\right)\right|$ will be small.

We then aggregate over all height pairs to get an overall coherence score for sensor *m* at location *u*. We define

(26) $$S_{m}\left(u\right)=\sum_{(q,q^{\prime })\in H\times H}|\;C_{m}\;\left(u;\;q,q^{\prime }\right)\;|,$$

where $S_{m}\left(u\right)$ in Equation (26) essentially sums up the magnitudes of the coherence metrics over all combinations of assumed heights for sensor *m* and the reference. One can view $S_{m}\left(u\right)$ as a sensor-specific “likelihood” or scoring function indicating how plausible it is, based on sensor *m*’s data (in comparison to the reference), that the transmitter is located at *u*. If *u* is incorrect, we expect that no choice of $\left(q,q^{\prime }\right)$ will yield a consistently strong coherence, so the sum of magnitudes will remain low. If *u* is near the true transmitter location, then there should be at least one pair $\left(q,q^{\prime }\right)$ (the one corresponding to the actual propagation scenario) that yields a strong coherent alignment between *m* and $r^{★}$, thereby giving a large contribution to the sum.

Joint spectrum computation and maximization: Finally, we combine the per-sensor scores $S_{m}\left(u\right)$ from all the selected sensors m∈M (i.e., all the ordinary sensors, excluding the reference) to form a joint spatial spectrum F(u). We define

(27) $$F\left(u\right)=\prod_{m\in M}S_{m}\left(u\right).$$

In Equation (27), F(u) is the product of the scores from each of the K−1 sensors, excluding the reference. The idea behind using a product is that we want *u* to be a strong candidate only if *all* the selected sensors have good alignment (high coherence) when the transmitter is hypothesized to be at *u*. If even one sensor has very low $S_{m}\left(u\right)$, the product will be zero or very small, indicating *u* is unlikely because it fails to match the data at that sensor. Thus, F(u) will peak at the location *u* that best explains the timing across the entire network of sensors simultaneously. Once we have computed F(u) for all candidate points u∈U, we pick the maximizer as our estimated source location:

(28) $$\hat{u}\;=\;\arg\max_{u\in U}\;F\left(u\right),$$

where the position $\hat{u}$ defined in Equation (28) is the output of Stage B’s coarse search. Often, it is beneficial to refine this estimate further. One common refinement technique is to take the initial estimate $\hat{u}$ and then perform a local search in its vicinity. For example, we can generate a small “cloud” of trial points around $\hat{u}$ by adding random perturbations (e.g., Gaussian-distributed offsets with a certain standard deviation forming a covariance matrix Σ) to $\hat{u}$. Let ${\left\{{\tilde{u}}^{\left(p\right)}\right\}}_{p=1}^{P_{\mathrm{loc}}}$ denote these local trial positions (with ${\tilde{u}}^{\left(1\right)}=\hat{u}$ possibly included). We then evaluate F(u) on this smaller set of points and pick the best among them, which gives us an updated $\hat{u}$. Because this second search is only over a local neighborhood (and we already have a good starting point), we can afford to make the perturbations fine-grained (small) and use more points $P_{\mathrm{loc}}$ if needed, to hone in on a more precise estimate. This two-stage grid approach (a coarse global search followed by a finer local search around the best point) improves both accuracy and robustness without incurring the full cost of a uniformly dense global search.

Optionally, once we have a final location estimate $\hat{u}$, we might also be interested in the likely virtual reflection heights for each path. The algorithm naturally provides a way to estimate these as well: for each sensor m∈M and even for the reference $r^{★}$, we can find which height h∈H best explains that sensor’s data given the source is at $\hat{u}$. One simple approach is to choose

(29) $${\hat{h}}_{m}\;\in \;\arg\max_{h\in H}\;\sum_{q^{\prime }\in H}|\;C_{m}\;\left(\hat{u};\;h,\;q^{\prime }\right)\;|\;,\;\mathrm{for}\;\mathrm{each}\;m\in M\cup \left\{r^{★}\right\}\;.$$

In other words, for each sensor *m*, we consider all coherence values $C_{m}\left(\hat{u};h,q^{\prime }\right)$ where the height for sensor *m* is fixed to *h* and the reference’s height $h^{\left(q^{\prime }\right)}$ varies over H. We sum the magnitudes $|C_{m}|$ over all $q^{\prime }$ to get a total coherence score for sensor *m* assuming a particular height *h*. We then pick the height that maximizes this score as ${\hat{h}}_{m}$. This ${\hat{h}}_{m}$ can be interpreted as the algorithm’s estimate of the effective ionospheric reflection height for the path to sensor *m*. It is worth noting that this height estimate is a byproduct and not required for localization; however, it can be valuable for understanding the propagation scenario or for further processing.

To provide a concise guide for implementation, the following Algorithm 2 summarizes the above RSS-based direct Geolocation procedure.
**Algorithm 2** Stage B: RSS-based Direct Geolocation on $S_{\mathrm{sel}}$**Input:** $S_{\mathrm{sel}}$ with reference $r^{★}$; streams $R_{m}\left(k\right)$ for $m\in S_{\mathrm{sel}}$, k∈K; particles U, H; stabilizer ε.2:**for** 
u∈U 
**do**   **for** m∈M **do**4:     $S_{m}\left(u\right)\leftarrow 0$.     **for** $(q,q^{\prime })\in H\times H$ **do**6:        compute $\Delta \tau_{m}^{\left(q,q^{\prime }\right)}\left(u\right)$ via (24);        accumulate $C_{m}\left(u;\left(q,q^{\prime }\right)\right)$ via (25);8:        $S_{m}\left(u\right)\leftarrow S_{m}\left(u\right)+\left|C_{m}\left(u;\left(q,q^{\prime }\right)\right)\right|$.     **end for**10:   **end for**   $F\left(u\right)\leftarrow \prod_{m\in M}S_{m}\left(u\right)$.12:**end for**$\hat{u}\leftarrow \arg\max_{u\in U}F\left(u\right)$; optionally refine locally around $\hat{u}$ and update $\hat{u}$.14:**Output:** $\hat{u}$ (and, optionally, $\left\{{\hat{h}}_{m}\right\}$ via (29)).

From the pseudocode above, the dominant computational cost in Stage B comes from evaluating the spatial spectrum F(u) on the joint grid U×H using the selected receiver subset *K*. For each candidate location u∈U and each receiver $m\in S_{\mathrm{sel}}$, the algorithm loops over all height pairs $(q,q^{\prime })\in H\times H$ to compute delay hypotheses and coherence terms and then aggregates them into $S_{m}\left(u\right)$ and F(u). In other words, the overall complexity grows approximately linearly with the number of candidate locations |U| and selected receivers *K*, and quadratically with the number of virtual-height candidates |H| through the H×H loop. In the emergency eVTOL scenarios considered in Section 5, Stage A restricts *K* to only five receivers and confines *U* to a small search rectangle in the region of interest, while *H* contains only a few tens of virtual-height candidates consistent with Section 2, so the grid search remains numerically stable and computationally tractable in our experiments.

## 5. Simulation and Experimental Results

### 5.1. Simulation Environment Configuration

The simulation scenario’s geographic range is set to 10° N–60° N latitude and 70° E–140° E longitude. Eight candidate HF receivers are deployed within this region and indexed by i=1,…,8; their site names and geographic coordinates are summarized in Table 1. We consider S=8 candidate receivers in total, and let *K* denote the number of receivers selected in each simulation run. In all simulations we set K=5, so Stage A selects 5 out of the 8 candidates according to the proposed receiver-selection strategy. Propagation uses a skywave equivalent single-hop model. The ground grid *U* is constructed as a regular latitude–longitude mesh over the region of interest with a step size of 0.25°, and the virtual-height grid *H* uniformly samples the physically reasonable range 80–500km with a 20km spacing, consistent with the virtual-height range discussed in Section 2. The transmitted signal is AM with a 5 kHz bandwidth and a modulation index of 0.75. Each receiver samples synchronously at a 10 kHz sampling rate, recording 15,000 baseband samples per pass. SNR is defined as $\mathrm{SNR}=10\log_{10}\;\left(\frac{P_{v}}{\sigma^{2}}\right)$ and is evaluated at discrete points ranging from −10:5:20dB. Each simulation starts from a unified geometry and waveform configuration, place a source in the area of interest, and propagate the baseband signal to eight candidate receivers using a single-hop virtual-height HF model to obtain band-limited observations. AWGN is then added to the same observations to meet the target SNR (definition and values are given in the “Parameter Settings” section), and random seeds are fixed for reproducibility. Stage A performs receiver selection on the same candidate set (both the unknown-bias route and the bias-prior robust route), yielding the subset for this run; Stage B evaluates the RSS objective over the same geographic grid *U* and height set *H* and returns $\hat{u}$ (and $\hat{h}$).

All TDOA-based baseline algorithms in Section 5.4 operate on exactly the same observations, receiver subsets, grids, and stopping criteria; if a baseline requires TDOA/cross-correlation, these intermediates are estimated from the same data with no oracle information. Runs are repeated independently across day/night and month conditions, and we aggregate position errors to report RMSE; hyperparameter tuning is separated from test months to prevent leakage, ensuring a fair comparison to ReSL–RSS. All simulation results in Section 5 are obtained from repeated runs of this unified configuration: for each SNR value and each receiver subset, one set of synthetic HF measurements is generated and then processed by ReSL-RSS, Random-K-RSS and all baseline algorithms using the same geolocation error metric. The main HF signal, propagation, and RSS grid parameters used throughout Section 5 are also summarized in Table 2 for clarity.

In addition, we evaluated the computational cost of the complete two-stage ReSL-RSS pipeline using our MATLAB 2024B prototype. On a standard desktop PC with an Intel Core i7 CPU 3.2 GHz and 16 GB of RAM, a single Stage B run under with K=5 selected receivers and the grids *U* and *H* defined above requires about 0.5 s, and the optional local refinement around the maximizer adds approximately 0.1 s. For HF data recording in our actual tests, one complete execution of Stage A followed by Stage B takes approximately 0.6 s from the arrival of the synchronized I/Q data at the processing center to the final location estimate. This latency is shorter than the data-window length, indicating that, for our prototype HF-sensor system, a usable position estimate can be generated within about 0.6 s from data acquisition to availability at the operation console. In real emergency operations, however, the time from having this location estimate to taking concrete rescue or air-traffic-control actions will additionally include decision-making, coordination and safety procedures at the operation center, so the operational response time will be longer than the localization latency reported here.

For the RSS stage, several implementation parameters are configured as follows. The DFT length $K_{f}$ is set equal to the number of baseband samples, and the frequency set $K\in (0,\ldots ,K_{f}-1)$ is chosen as the contiguous group of bins that cover the occupied 5 kHz AM band, excluding bins where the reference spectrum falls more than 20 dB below its peak. The stabilizer ε in (25) is defined as a small fraction of the average reference power over *K*,

(30) $$\varepsilon =10^{-3}\;\frac{1}{\left|K\right|}\sum_{k\in K}{\left|R_{r^{★}}\left(k\right)\right|}^{2}\;,$$

which prevents division by zero and improves numerical stability without dominating the denominator.

To evaluate the robustness of the proposed ReSL–RSS framework against ionospheric variability and seasonal/daytime effects, we carried out an additional set of robustness experiments over a full year. Using the same HF geometry, we considered two mid-latitude propagation conditions (daytime and nighttime) and twelve calendar months.

**Figure 2:**
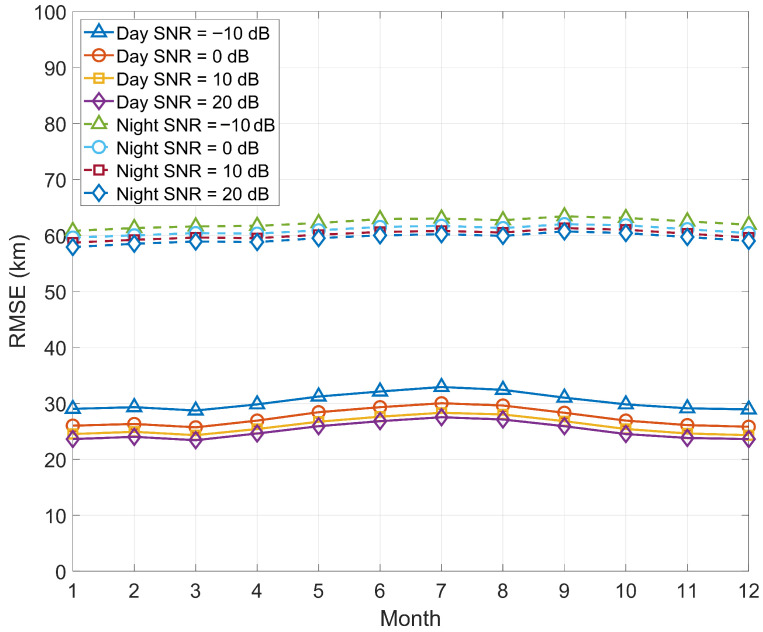
RMSE of HF source position estimation in the daytime and nighttime in different seasons.

Figure 2 shows that increasing SNR systematically reduces RMSE, a trend that is more pronounced during the day. The nighttime curves are nearly parallel and close to each other, indicating that uncertainty in nighttime HF propagation dominates the error, and simply increasing SNR has limited benefits. For example, in July, the daytime RMSE decreases from ≈33km at SNR=−10dB to ≈27km at SNR=20dB; whereas the nighttime RMSE only decreases from ≈63km to ≈61km over the same range. Across months, the fluctuations of the daytime SNR–RMSE curves are ≈3–5km, and at night ≈2–3km; there is a slight increase from June to August, followed by a decrease from October to December, consistent with the empirical understanding that increased ionospheric activity in summer exacerbates the uncertainty of HF paths.

To demonstrate the effectiveness of our proposed ReSL-RSS algorithm, we will conduct the following experiments. First, we will compare RSS geolocation using the receiver selection method in stage A with random receivers selection. Second, to demonstrate that stage A selects the optimal receiver, we permutate all the cases of candidate receivers. RSS geolocation experiments were conducted in each receiver case to compare the geolocation errors. Finally, after selecting the optimal receiver, we will compare it with traditional geolocation algorithms and geolocation algorithms from recent references.

### 5.2. Comparison of ReSL-RSS with the Baseline Random-K-RSS

The two receiver selection scenarios of ReSL-RSS, Case 1 and Case 2, are com-pared with random receiver selection followed by RSS geolocation (Random-K-RSS).

**Figure 3:**
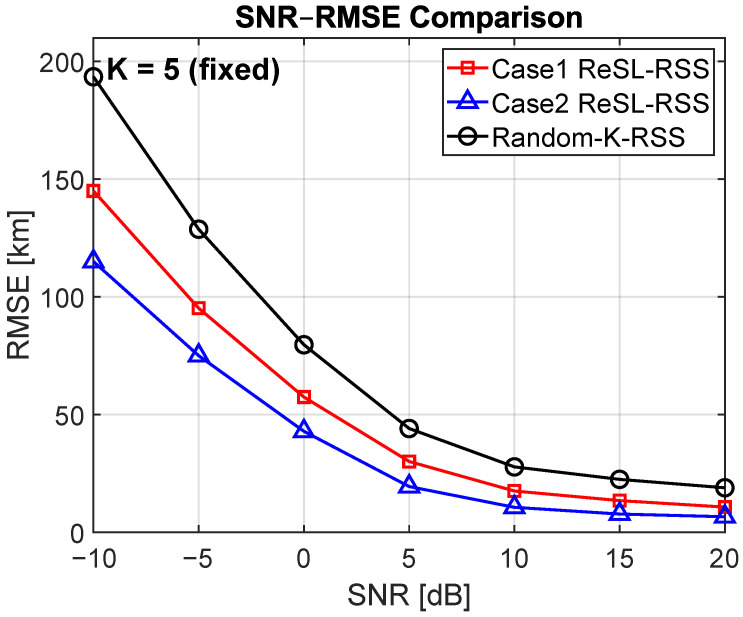
RMSE versus SNR for a fixed set of K = 5 receivers.

Figure 3 shows the SNR-RMSE curves for a fixed K = 5. Cases 1 and 2 consistently outperform the Random-K algorithm, with the gap being most pronounced at low SNRs. For example, at a SNR of −10dB, the RMSEs for Random-K, Case 1, and Case 2 are approximately 192 km, 145 km, and 115 km, respectively. At a SNR of 0 dB, the RMSEs are approximately 80 km, 57 km, and 43 km, respectively, representing reductions of approximately 31% and 44% relative to Random-K. As the SNR increases, the three curves approach the noise-limited region, narrowing the gap. However, even at 20 dB, the differences remain constant and do not cross.

**Figure 4:**
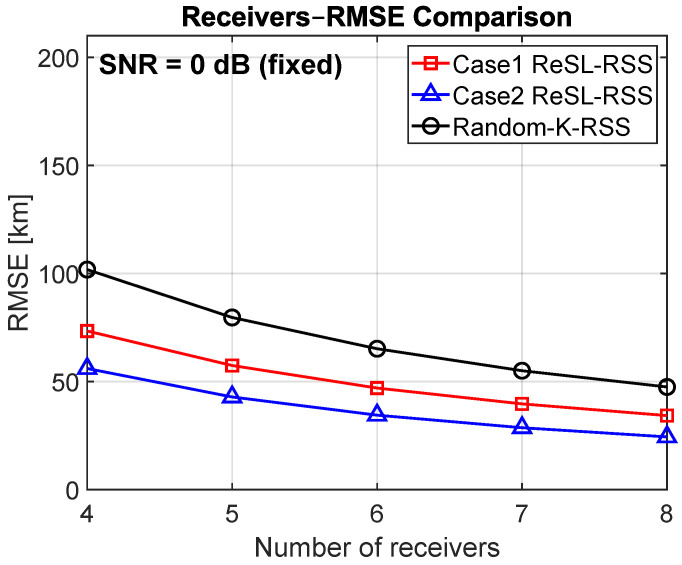
RMSE versus number of receivers for SNR = 0 dB (fixed).

Figure 4 shows the relationship between RMSE and the number of receivers for a fixed SNR of 0 dB. Taking K = 5 as the representative point, the RMSEs for random K, Case 1, and Case 2 are approximately 80 km, 57 km, and 43 km, respectively (corresponding to Figure 3). At K = 8, the RMSEs further decrease to approximately 47 km, 35 km, and 25 km. The curves are closer, but still do not intersect. This confirms that the benefits come not only from using more receivers, but also from the receiver selection criteria in stage A, Case 1 selects based on observation information, while Case 2 also incorporates a perreceiver bias prior, effectively suppressing the risk of systematic bias. Consequently, Case 2 generally performs better than Case 1, especially at low SNRs.

### 5.3. The Selection of the Top 5 Receivers

To demonstrate the importance of prior receiver selection, we select five out of eight candidate receivers. A complete list of all five-out-of-eight ($C_{5}^{8}=56$) combinations is presented. About 200 simulations were conducted under the same SNR and RSS geolocation, and the geolocation error distribution for each subset S was calculated.

**Figure 5:**
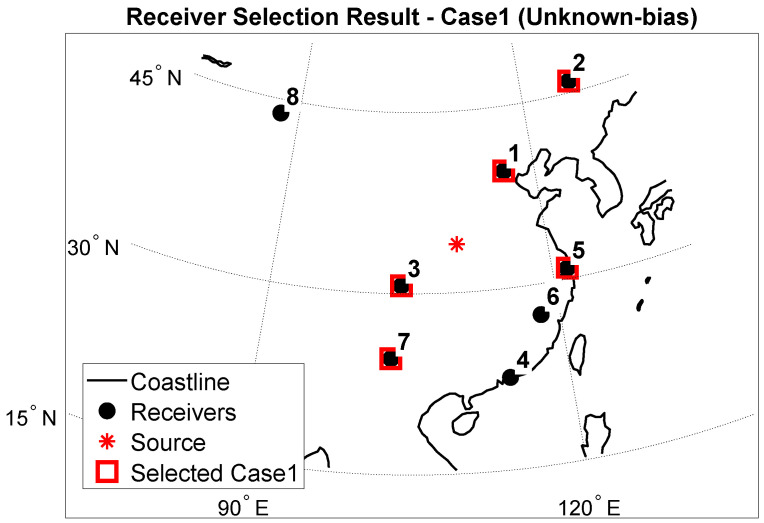
Receiver selection result—Case1.

**Figure 6:**
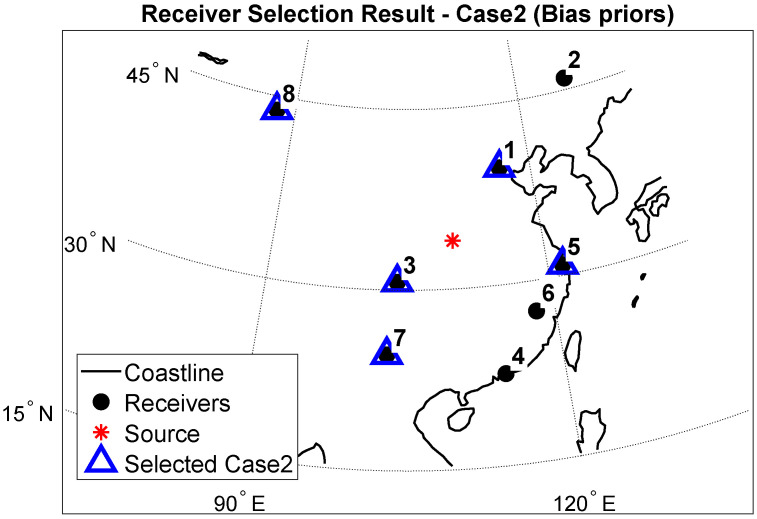
Receiver selection result—Case2.

Figure 5 and Figure 6 show the spatial distribution of the best five receivers for Case 1 and Case 2. The two optimal sets differ, Case 2 favors a westward, longer eastwest baseline, while Case 1 favors including receivers at higher latitudes in the north to enhance north-south geometry. This is due to different objectives. Case 1, under the unknown bias setting, minimizes the Schur complement-based CRLB, thereby emphasizing the reduction of geometric dilution in weak directions. Case 2 uses prior information as a parameter to form a very strong information matrix. Therefore, the global optimal value may differ.

Table 3 and Table 4 summarize the relative RMSE gaps to the optimal subset (“Gap to best [%]”) when SNR = 0. The optimal set for Case 1 is [2, 1, 5, 3, 7], corresponding to receivers (Harbin, Beijing, Shanghai, Chengdu, Kunming) according to the receiver numbering in Table 1 (receiver 2: Harbin; receiver 1: Beijing; receiver 5: Shanghai; receiver 3: Chengdu; receiver 7: Kunming) with an average RMSE of 57.42 km. The optimal set for Case 2 is [8, 1, 3, 7, 5], corresponding to (Urumqi, Beijing, Chengdu, Kunming, Shanghai) under the same receiver-numbering convention in Table 1, with an average RMSE of 42.86 km. This data corresponds to those in Section 5.2. Comprehensive analysis shows that, under the same conditions, both Case 1 and Case 2, using receiver selection followed by RSS geolocation, outperforms the random-receiver (Random-K) selection followed by RSS geolocation.

### 5.4. Comparison of ReSL-RSS with Traditional NLOS Algorithms

The optimal five receiver combinations were determined using stage A of ReSL-RSS. The optimal combination for Case 1 was [2, 1, 5, 3, 7], and the optimal combination for Case 2 was [8, 1, 3, 7, 5]. For a fair comparison, these optimal five-receiver subsets are then kept fixed, and all methods are evaluated under exactly the same receiver geometry in each case. After fixing the optimal receivers for Case 1 and Case 2, we compared the algorithm proposed in this article with some geographic positioning methods in the references: GA-TDOA [35], HSCGP [36], GPGD-TDOA [37], TOA-TDOA [38], IOD [39] and TDOA-CHAN [40]. All of these baselines are TDOA-based positioning methods, so applying them under the same five receivers and propagation conditions allows a direct comparison focused on the positioning algorithms themselves rather than on different receiver layouts. Under the five optimal receivers [2, 1, 5, 3, 7] selected in Case 1, this five-receiver set is kept fixed, and Case 1 simulations are run for ReSL-RSS and for all of the above geolocation algorithms. Similarly, under the five optimal receivers [8, 1, 3, 7, 5] selected in Case 2, this five-receiver set is fixed and used by ReSL-RSS and all baseline methods in the Case 2 simulations.

The empirical cumulative distribution function (CDF) of the absolute geolocation errors for the two stage A selection schemes is plotted. CDF allow us to compare the entire error distribution, not just a single average.

**Figure 7:**
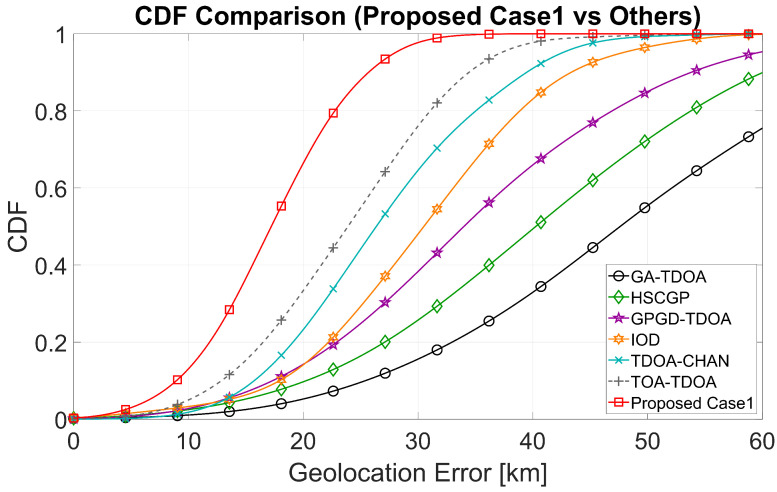
CDF of the error distribution (Comparison of Case 1 with other algorithms).

**Figure 8:**
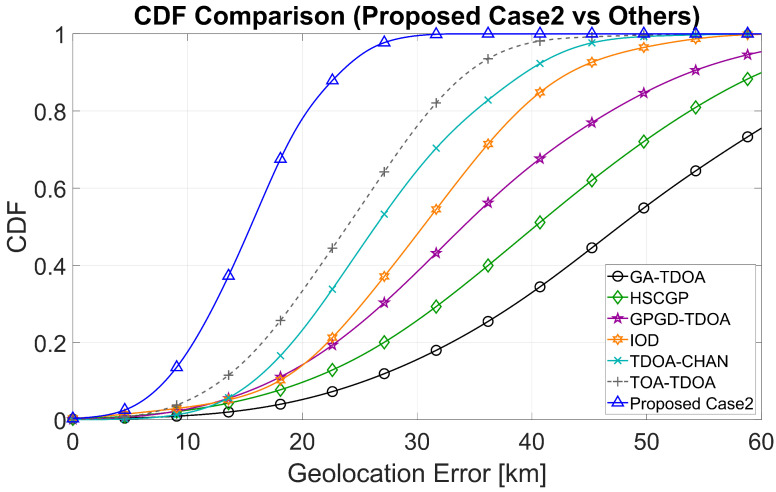
CDF of the error distribution (Comparison of Case 2 with other algorithms).

In Figure 7, the ReSL-RSS curve (red) is located at the extreme left/top for most quantiles. Near the median (CDF ≈0.5), ReSL-RSS is approximately 19–21km, significantly lower than the other algorithms. At high quantiles, ReSL-RSS still reaches the minimum threshold. In Figure 8, the ReSL-RSS curve (blue) shifts further to the left relative to Case 1; the median drops to approximately 16–18km, still at the lowest level. A significant gap persists at high quantiles, indicating improved error suppression after incorporating bias risk information in Stage A.

Box plots of geolocation error visually demonstrate the error level and severity of each method. The horizontal line within the box represents the median, the box represents the range of the middle half of the results, and the upper whiskers and scatter plots represent larger errors.

**Figure 9:**
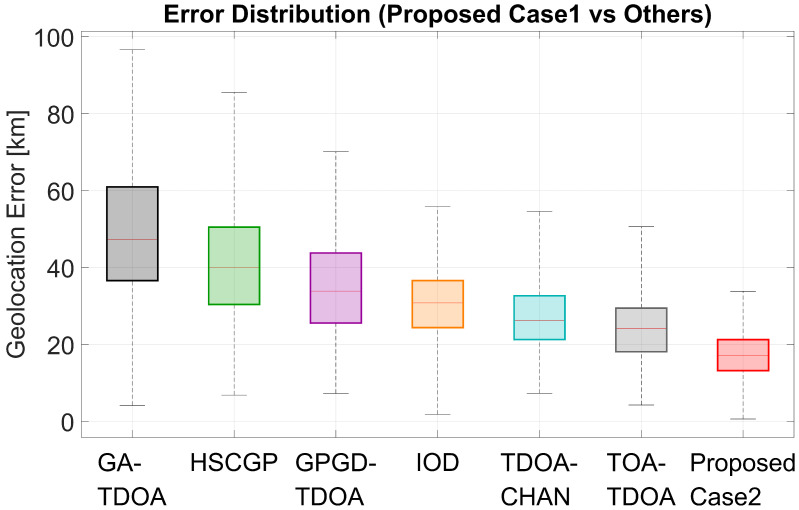
Boxplot of Case 1 compared with other algorithms.

**Figure 10:**
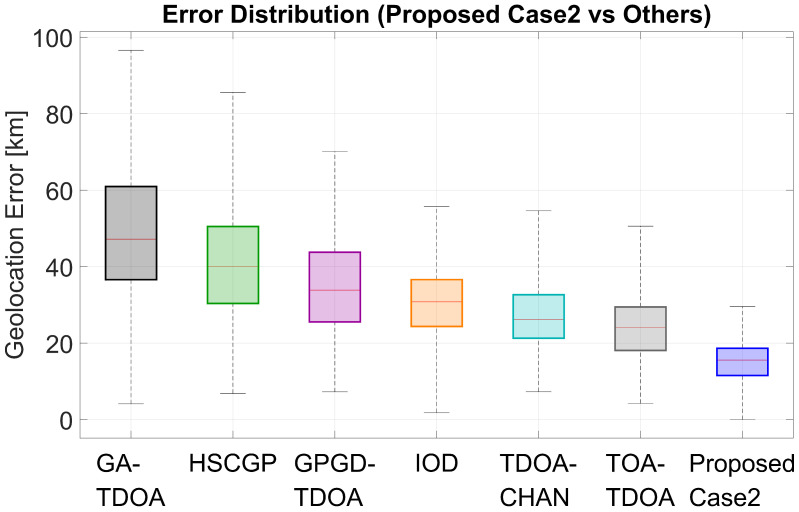
Boxplot of Case 2 compared with other algorithms.

For Case 1, Figure 9 shows that after using the optimal five receivers for that case, ReSL-RSS achieves the lowest and narrowest box, the lowest median, and fewer upper whiskers and outliers, indicating smaller errors, less fluctuation, and fewer instances of large errors. For Case 2, as shown in Figure 10, the introduction of the bias prior causes the ReSL-RSS bin to shift further downward, and narrow compared to Case 1. The baseline method also declines overall, but the relative ranking remains largely unchanged, with the gap with ReSL-RSS remaining clear at the media and above quantiles. Overall, under both receiver selection settings, ReSL-RSS more often achieves smaller errors and less frequently experiences large errors.

**Figure 11:**
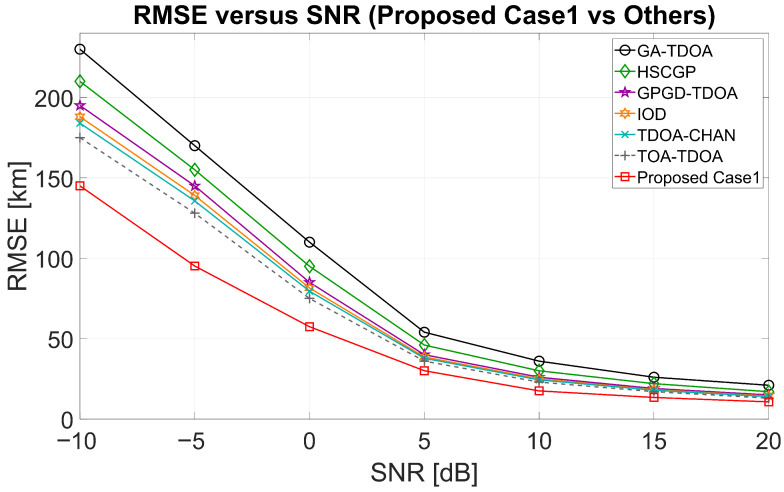
RMSE–SNR curves (Case 1 compared with other algorithms).

**Figure 12:**
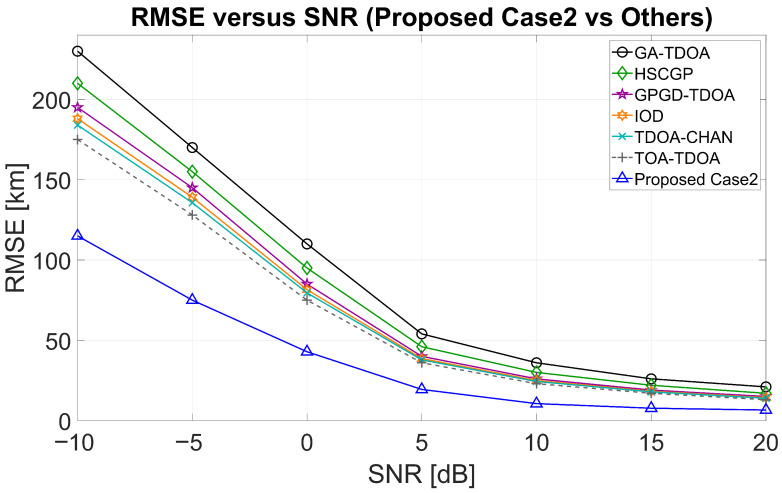
RMSE–SNR curves (Case 2 compared with other algorithms).

Figure 11 shows the RMSE curves of ReSL-RSS and other algorithms as SNR changes, with the optimal five receivers selected in Case 1 remaining unchanged. ReSL-RSS (red line) maintains its lowest value across the entire SNR range, with the most significant difference at low SNR. As SNR increases, the RMSE of all methods decreases and gradually converges. The gap narrows significantly in the high SNR range, but no overlap occurs. ReSL-RSS is generally lower. Figure 12 shows the RMSE curves of ReSL-RSS and other algorithms like SNR changes, with the optimal five receivers selected in Case 2 remaining unchanged. After using a bias prior for receiver selection, ReSL-RSS (blue line) shifts further downward overall, exhibiting lower RMSE than Case 1 at all SNR points. This improvement remains clearly visible in the low SNR range but converges and reaches its lowest value at high SNRs. The curve values for Cases 1 and 2 are largely consistent with the simulation curves in Section 5.2. Together, these two figures demonstrate that ReSL-RSS achieves lower geolocation error than other algorithms, with the advantage becoming even more pronounced after introducing a bias prior.

**Figure 13:**
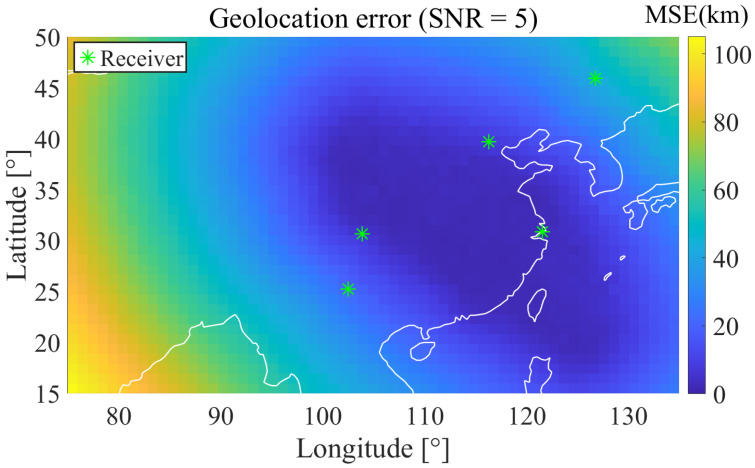
Geolocation error heatmap (Case 1 ReSL-RSS optimal 5-receiver SNR = 5 dB).

**Figure 14:**
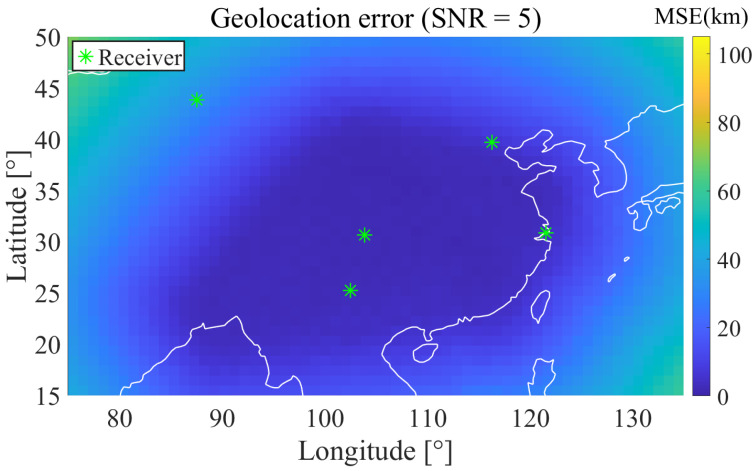
Geolocation error heatmap (Case 2 ReSL-RSS optimal 5-receiver SNR = 5 dB).

Figure 13 shows the geolocation error distribution of the five best receivers selected in Case 1 under their respective ReSL-RSS conditions, with an SNR of 5 dB. Green asterisks represent receivers. Low errors are concentrated in the longitude region around 108° E–118° E and 30°–40° N. A prominent high-error band appears in the western region between 80°–90° E on the left. This is related to the insufficient east-west baseline length of the array selected in Case 1. Geometric information has low eigenvalues in the east-west direction, resulting in a large CRLB gradient in this direction that gradually increases from east to west. Figure 14 shows the geolocation error distribution of the five best receivers selected in Case 2 under their respective ReSL-RSS conditions, with an SNR of 5 dB. Due to the introduction of a bias prior, the east-west baseline is significantly elongated. Compared with Case 1, the low-error zone expands significantly westward and northward, while the high-error band in the west is squeezed shallower and its contours are more rounded, indicating reduced anisotropy and more balanced geometric conditions. This is consistent with the statistical conclusion proposed previously, under biased prior conditions, Case 2 can suppress systematic errors more effectively than Case 1.

Overall, the above series of simulation experiments demonstrate a complete chain of evidence, assuming the candidate receivers remain fixed and all other conditions remain the same. First, using the same RSS geolocation after stage A receiver selection and random receiver selection, the results show significant differences in error, with ReSL-RSS outperforming RSS geolocation after random receiver selection. To further demonstrate the optimal receiver selection in stage A, multiple simulations were conducted, comparing all possible geolocation errors. Finally, under the condition of a fixed optimal receiver, since the optimal receivers differ between Case 1 and Case 2, we compared the results with other algorithms using the optimal receiver in Case 1 and the optimal receiver in Case 2. The results demonstrate that ReSL-RSS outperforms the other algorithms, with Case 2 outperforming Case 1. Numerical results are essentially consistent with those obtained in the first stage, using the same RSS geolocation after stage A receiver selection and random receiver selection. Initial receiver selection improves geometric observability and reduces bias risk, while RSS geolocation further reduces the average error and converges tail risk.

### 5.5. Experimental Results

Based on the above simulation, we conducted actual measurement on the ReSL-RSS algorithm. The geolocation system consists of eight HF signal receivers with GPS time synchronization, deployed at the same sites as the eight candidate receivers described in Section 5.1. Each receiver uses baseband I/Q sampling to synchronously capture the target signal. The I/Q sequences are synchronously recorded at all receivers. Each receiving site is equipped with a short-wave vertical monopole antenna and a GPS-based software-defined radio (SDR) front-end. The eight receivers are deployed at Beijing, Harbin, Shanghai, Wuyishan, Chengdu, Kunming, Shenzhen, and Urumqi, i.e., at the same geographic locations as the candidate sensors listed in Section 5.1, and they record synchronized complex baseband I/Q data. The receivers are tuned to the same HF channel and use the AM signal format, 5 kHz occupied bandwidth, and 10 kHz sampling rate specified in the simulation configuration of Section 5.1, so that the field trial shares a consistent waveform and bandwidth with the synthetic data. For baseline methods and data consistency checking, we further process these I/Q sequences to obtain time delay estimation (TDE) results with respect to the reference receiver at Wuyishan. It should be emphasized that the proposed ReSL-RSS algorithm itself still operates directly on the synchronized I/Q samples, as described in Section 4, without using the TDEs as inputs. The high-frequency source for this experiment is geolocation in Xi’an $\left(108^{\circ }61^{\prime }\;E,\;34^{\circ }37^{\prime }\;N\right)$. This transmitter is implemented as a fixed ground-based HF beacon and serves as a surrogate distress source for an eVTOL platform that has lost communication.

**Figure 15:**
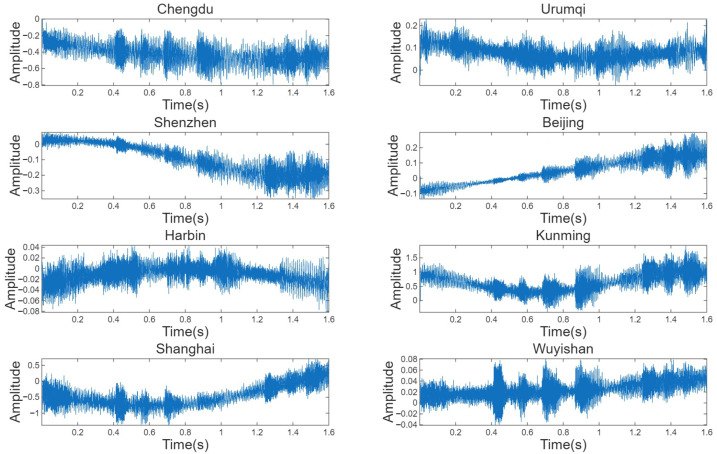
23 September 2024, 1.6 s measured amplitude record of each receiver.

Figure 15 shows 1.6-s amplitude records simultaneously observed on 23 September 2024, at eight receivers in Chengdu, Urumqi, Shenzhen, Beijing, Harbin, Kunming, Shanghai, and Wuyishan. The envelopes in Beijing and Shanghai are relatively stable, with minimal short-term fluctuations. Shenzhen and Kunming maintain high effective amplitudes and short-duration deep fades. Although Wuyishan exhibits some sudden fluctuations, the correlation peaks are clear, making it suitable for delay estimation. In contrast, the envelopes in Chengdu and Harbin exhibit more pronounced drift and larger fluctuations, while the amplitude oscillations in the Urumqi segment are stronger. Based on a comprehensive evaluation of these measurement characteristics, ReSL-RSS identified five receivers in Shenzhen, Beijing, Kunming, Shanghai, and Wuyishan as the subset for this geolocation. This subset was used for geolocation, we apply ReSL-RSS and all TDOA-based baseline algorithms to the measured I/Q data and compare their geolocation performance.

Table 5 lists the arrival order and path differences of each receiver relative to the reference receiver “Wuyishan.” The signs are consistent with the geometric relationship, Beijing (−220.37 km) is negative, indicating that they are closer to the signal source and arrive earlier than the reference receiver, Shanghai (+80.38 km), Kunming (+186.24 km), and Shenzhen (+271.44 km) are positive, indicating that they arrive later. Converting the path differences to delays, the path differences for Beijing, Shanghai, Kunming, and Shenzhen are approximately −0.735 ms, +0.268 ms, +0.621 ms, and +0.905 ms, respectively.

**Figure 16:**
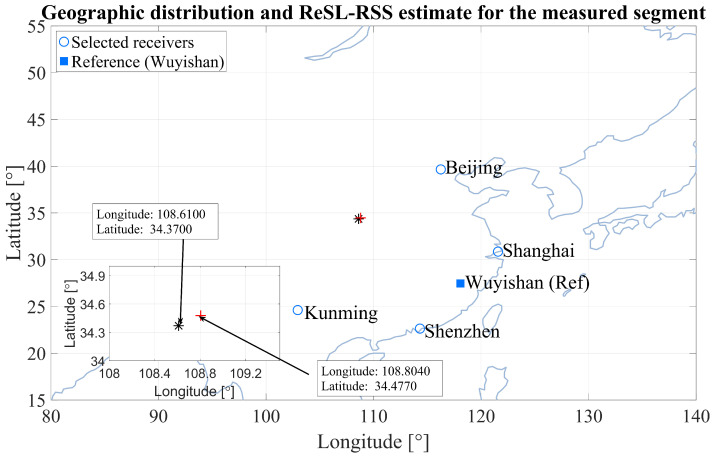
Geographic locations of the receivers and the HF source and the location estimates of the HF source.

Figure 16 shows the measured receiver distribution and the ReSL-RSS geolocation results. The five receivers selected above with good signal quality are used, and the reference receiver is fixed at Wuyishan (blue square). A zoom window is shown in the lower left corner of the figure, marking the true source location (black *, $108^{\circ }61^{\prime }$ E, $34^{\circ }37^{\prime }$ N) and the ReSL-RSS estimate (red “+”, 108°80′40″ E, 34°47′7″ N) for accurate comparison. The zoom window indicates that the estimated location is slightly southwest of the true location, differing by approximately $0.194^{\circ }$ in longitude and $0.107^{\circ }$ in latitude, resulting in a geolocation error of approximately 21.71 km.

Finally, we compared the proposed ReSL-RSS with various geolocation algorithms. Table 6 summarizes the average geolocation error of each method, as well as the percentage of excess error relative to ReSL-RSS, under the same data segment and evaluation settings. ReSL-RSS has the lowest average error, followed by TOA-TDOA and TDOA-CHAN, while HSCGP and GA-TDOA have the highest average errors. This is consistent with the simulation results. Under the same locator and settings, the receiver selection-before-geolocation approach significantly reduces error.

## 6. Conclusions

This paper proposes a two-stage ReSL-RSS framework for HF emergency geolocation in eVTOL loss-of-connection scenarios. Stage A selects receivers based on geometric observability, while stage B performs RSS geolocation. Simulations and field measurements were conducted using this unified design. In simulations, we first compared RSS geolocation with random receivers. Then, after multiple experiments, we selected the top five receivers. Finally, we conducted comparative experiments using fixed receivers. In field measurements, we selected five receivers with the best signal quality based on the measured receivers for geolocation and comparison. The results show that the proposed method of selecting a receiver first and then performing geolocation is more effective than other direct geolocation algorithms. Overall, the proposed ReSL-RSS framework appears to be a promising and reproducible technical solution for HF emergency geolocation, and the field trial provides initial evidence of its effectiveness. Nevertheless, comprehensive operational validation with real eVTOL platforms and dedicated emergency scenarios will be an important direction for future work.

## Figures and Tables

**Table 1:** Candidate receivers.

Receiver Number	Site	Latitude (°N)	Longitude (°E)
1	Beijing	$39^{\circ }66^{\prime }\;N$	$116^{\circ }26^{\prime }\;E$
2	Harbin	$45^{\circ }94^{\prime }\;N$	$126^{\circ }79^{\prime }\;E$
3	Chengdu	$30^{\circ }66^{\prime }\;N$	$103^{\circ }88^{\prime }\;E$
4	Shenzhen	$22^{\circ }64^{\prime }\;N$	$114^{\circ }32^{\prime }\;E$
5	Shanghai	$30^{\circ }87^{\prime }\;N$	$121^{\circ }58^{\prime }\;E$
6	Wuyishan	$27^{\circ }46^{\prime }\;N$	$118^{\circ }08^{\prime }\;E$
7	Kunming	$24^{\circ }62^{\prime }\;N$	$102^{\circ }95^{\prime }\;E$
8	Urumqi	$43^{\circ }85^{\prime }\;N$	$87^{\circ }54^{\prime }\;E$

**Table 2:** Simulation parameters.

Parameter	Value and Description
Geographic region	$10^{\circ }\;N–60^{\circ }\;N$ latitude, $70^{\circ }\;E–140^{\circ }\;E$ longitude
Number of candidate receivers *S*	S=8
Number of selected receivers *K*	K=5 receivers selected by Stage A in each simulation run
Propagation model	Single-hop equivalent HF skywave; virtual height uniformly sampled between 80 and 500km
Transmitted signal	AM waveform with 5kHz bandwidth and modulation index 0.75
Sampling rate and record length	10kHz sampling, 15,000 complex baseband samples per receiver per pass
SNR definition and range	$\mathrm{SNR}=10\log_{10}\;\left(P_{v}/\sigma^{2}\right)$; evaluated at −10,−5,0,5,10,15,20dB
Ground search grid *U* (RSS stage)	Latitude–longitude mesh over the region of interest with $0.25^{\circ }$ spacing
Virtual-height grid *H* (RSS stage)	Heights from 80km to 500km with 20km spacing
DFT length $K_{f}$	$K_{f}$ equal to the number of baseband samples $\left(K_{f}=15,000\right)$
Frequency subset *K*	Contiguous bins covering the occupied 5kHz AM band, excluding bins whose reference spectrum is >20dB below the peak

**Table 3:** Case 1 (ReSL-RSS): 56 groups of 8 choose 5; receiver numbers follow the numbering in Table 1.

Receivers	RMSE_avg [km]	Gap vs. Best [%]	Receivers	RMSE_avg [km]	Gap to Best [%]
**2, 1, 5, 3, 7**	**57.42**	**0.0%**	2, 4, 5, 3, 7	59.68	+3.94%
2, 1, 5, 3, 6	58.56	+1.99%	2, 5, 3, 7, 8	59.95	+4.41%
1, 2, 4, 6, 8	61.33	+6.81%	1, 2, 4, 6, 7	62.97	+9.67%
1, 2, 4, 5, 6	63.84	+11.18%	1, 2, 5, 7, 8	65.18	+13.51%
1, 2, 3, 4, 5	65.72	+14.45%	1, 2, 3, 6, 8	67.55	+17.64%
2, 4, 5, 6, 7	68.31	+18.97%	3, 4, 5, 6, 7	71.33	+24.23%
2, 3, 5, 6, 8	69.95	+21.82%	3, 4, 5, 6, 8	74.89	+30.42%
1, 2, 4, 7, 8	72.11	+25.58%	1, 2, 6, 7, 8	78.17	+36.14%
1, 2, 3, 7, 8	74.32	+29.43%	1, 3, 4, 7, 8	81.56	+42.04%
1, 3, 5, 6, 8	76.61	+33.42%	1, 4, 5, 6, 8	82.23	+43.21%
2, 3, 4, 5, 8	78.94	+37.48%	2, 4, 6, 7, 8	84.72	+47.54%
2, 3, 4, 6, 8	81.13	+41.29%	1, 4, 5, 7, 8	85.33	+48.61%
1, 3, 4, 6, 8	83.41	+45.26%	2, 5, 6, 7, 8	86.66	+50.92%
1, 3, 6, 7, 8	85.74	+49.32%	2, 3, 4, 5, 6	89.21	+55.36%
2, 1, 6, 3, 7	60.13	+4.7%	2, 1, 5, 4, 7	59.93	+4.4%
1, 5, 3, 7, 8	60.45	+5.3%	2, 1, 4, 3, 7	61.39	+6.9%
1, 2, 5, 6, 7	61.81	+7.6%	1, 2, 3, 4, 8	64.05	+11.55%
1, 3, 4, 5, 8	63.34	+10.31%	1, 2, 3, 4, 6	67.52	+17.59%
2, 3, 4, 6, 7	65.19	+13.53%	2, 3, 5, 6, 7	69.97	+21.86%
1, 2, 5, 6, 8	66.67	+16.11%	2, 4, 5, 6, 8	73.75	+28.44%
1, 2, 4, 5, 8	67.27	+17.15%	3, 4, 5, 7, 8	77.10	+34.27%
1, 2, 3, 5, 8	67.89	+18.23%	1, 5, 6, 7, 8	80.41	+40.04%
1, 3, 5, 6, 7	69.59	+21.19%	1, 4, 5, 6, 7	83.28	+45.04%
2, 3, 6, 7, 8	72.36	+26.02%	3, 5, 6, 7, 8	87.36	+52.14%
3, 4, 6, 7, 8	77.32	+34.66%	4, 5, 6, 7, 8	88.94	+54.89%
1, 3, 4, 5, 7	79.62	+38.66%	2, 3, 4, 7, 8	90.36	+57.37%
1, 4, 6, 7, 8	82.36	+43.43%	1, 3, 4, 5, 6	92.26	+60.68%
1, 3, 4, 6, 7	85.51	+48.92%	2, 4, 5, 7, 8	94.75	+65.01%

Note: The “**Receivers**” list shows the receiver-number combinations obtained by selecting 5 out of 8 candidate receivers in this case (a total of 56 groups). **RMSE_avg [km]** is the simulated average RMSE (in km) when each receiver group localizes the same transmission source under SNR=0dB. **Gap to best [%]** is the relative increment with respect to the optimal combination in this table, defined as $\left({\mathrm{RMSE}}_{\mathrm{avg}}-{\mathrm{RMSE}}_{\mathrm{best}}\right)/{\mathrm{RMSE}}_{\mathrm{best}}\times 100\%$. Therefore, the Gap to best for the optimal combination is 0.0%.

**Table 4:** Case 2 (ReSL-RSS): 56 groups of 8 choose 5; receiver numbers follow the numbering in Table 1.

Receivers	RMSE_avg [km]	Gap vs. Best [%]	Receivers	RMSE_avg [km]	Gap to Best [%]
**8, 1, 3, 7, 5**	**42.86**	**0.0%**	8, 1, 4, 7, 5	45.58	+6.3%
8, 1, 3, 6, 5	44.87	+4.7%	8, 1, 4, 6, 5	46.35	+8.1%
1, 5, 6, 7, 8	46.62	+8.76%	1, 4, 6, 7, 8	47.17	+10.06%
1, 2, 4, 6, 8	49.45	+15.38%	1, 2, 3, 7, 8	49.76	+16.1%
1, 2, 4, 5, 8	50.93	+18.75%	1, 3, 4, 7, 8	51.28	+19.56%
2, 3, 4, 7, 8	52.63	+22.74%	2, 3, 4, 6, 8	53.05	+23.78%
1, 2, 3, 4, 7	54.59	+27.37%	1, 2, 3, 4, 6	54.72	+27.61%
1, 2, 3, 5, 6	55.94	+30.47%	1, 2, 3, 6, 7	56.44	+31.68%
1, 2, 5, 6, 7	57.58	+34.32%	1, 3, 4, 5, 7	58.01	+35.33%
1, 4, 5, 6, 7	59.32	+38.32%	1, 2, 4, 5, 6	59.72	+39.25%
2, 3, 4, 6, 7	61.03	+42.32%	2, 3, 5, 6, 7	61.39	+43.23%
1, 2, 5, 6, 8	62.67	+46.16%	1, 2, 5, 7, 8	63.14	+47.24%
2, 5, 6, 7, 8	64.32	+49.98%	3, 4, 5, 6, 8	64.73	+51.03%
3, 5, 6, 7, 8	66.91	+56.11%	4, 5, 6, 7, 8	70.35	+64.14%
8, 2, 4, 7, 5	44.75	+4.41%	8, 2, 3, 7, 5	46.78	+9.15%
8, 2, 3, 6, 5	46.79	+9.17%	8, 1, 3, 7, 6	49.15	+14.68%
1, 2, 4, 7, 8	50.23	+17.2%	1, 2, 6, 7, 8	52.11	+21.58%
1, 2, 3, 6, 8	51.26	+19.6%	1, 2, 3, 5, 8	53.29	+24.34%
1, 3, 4, 6, 8	52.37	+22.19%	1, 2, 4, 6, 7	55.67	+29.89%
2, 3, 4, 5, 8	54.69	+27.6%	1, 3, 5, 6, 7	57.36	+33.83%
1, 2, 3, 4, 5	55.51	+29.51%	2, 3, 4, 5, 7	58.68	+36.91%
1, 2, 4, 5, 7	57.94	+35.18%	3, 4, 5, 6, 7	60.54	+41.25%
1, 3, 4, 6, 7	59.81	+39.55%	2, 4, 5, 6, 8	62.78	+46.48%
2, 3, 4, 5, 6	60.27	+40.62%	3, 4, 6, 7, 8	63.96	+49.86%
2, 4, 5, 6, 7	62.33	+45.43%	1, 2, 3, 4, 8	64.53	+50.56%
3, 4, 5, 7, 8	64.87	+51.35%	1, 3, 4, 5, 6	66.78	+55.81%
1, 3, 4, 5, 8	65.66	+53.2%	2, 4, 6, 7, 8	70.23	+63.86%
1, 2, 3, 5, 7	69.35	+61.81%	2, 3, 6, 7, 8	72.61	+69.41%

Note: The meanings of “**Receivers**”, “**RMSE_avg [km]**”, and “**Gap to best [%]**” are the same as in Table 3.

**Table 5:** Delay estimation results of HF data.

Receiver (ref = Wuyishan)	Path Difference (km)	Delay (ms)
Beijing	−220.37	−0.735
Shanghai	+80.38	0.268
Kunming	+186.24	+0.621
Shenzhen	+271.44	+0.905
Wuyishan	0	0

**Table 6:** Measured geolocation performance.

Method	Avg. Error (km)	Gain vs. ReSL-RSS (%)
ReSL-RSS	21.71	0
TOA-TDOA	28.83	32.8
TDOA-CHAN	35.31	62.64
IOD	39.12	80.19
GPGD-TDOA	47.44	118.51
HSCGP	58.35	168.77
GA-TDOA	63.77	193.74

## Data Availability

The data presented in this study are available on request from the corresponding author. The data are not publicly available due to privacy reasons and the project funding requirement.
